# Effects of humic acid on Pb^2+^ adsorption onto polystyrene microplastics from spectroscopic analysis and site energy distribution analysis

**DOI:** 10.1038/s41598-022-12776-3

**Published:** 2022-05-27

**Authors:** Xiaotian Lu, Feng Zeng, Shuyin Wei, Rui Gao, Abliz Abdurahman, Hao Wang, Weiqian Liang

**Affiliations:** School of Chemistry, Sun Yat-Sen University, Guangzhou, 510275 China

**Keywords:** Environmental sciences, Chemistry

## Abstract

Microplastics (MPs), act as vectors of heavy metal pollutants in the environment, is of practical significance to study the adsorption process and mechanism on heavy metals. In this study, polystyrene microplastics (PSMPs) were used as model MPs to study the adsorption of Pb^2+^ on PSMPs and the effects of humic acid (HA) on the adsorption process. The results showed that HA promoted the adsorption of Pb^2+^ on PSMPs, and the higher the concentration of HA, the greater the adsorption of Pb^2+^. With the increase of pH value and decrease of ionic strength, the adsorption capacity of PSMPs for Pb^2+^ increased. The scanning electron microscope equipped with the energy dispersive spectroscope (SEM–EDS), fourier transform-infrared spectra (FT-IR) and X-ray photoelectron spectroscopy (XPS) analysis showed that Pb^2+^ could be adsorbed directly onto PSMPs and also indirectly by HA. The higher *K*_*SV*_ values in the PSMPs-HA-Pb^2+^ system than PSMPs-HA system by fluorescence analysis of HA suggested that HA acted as a bridging role in the adsorption of Pb^2+^ on PSMPs. The site energy distribution analysis further revealed that HA increased the average site energy *μ*(E^*^) and its standard deviation *σ*_e_^*^ of PSMPs by introducing more adsorption sites, thus enhanced the adsorption affinity of PSMPs. This study provided more thoughts and insights into the adsorption behavior and mechanism of MPs for Pb^2+^ in aquatic environments.

## Introduction

Microplastics (MPs) are defined as plastic fragments or particles with size less than 5 mm by the National Oceanic and Atmospheric Administration^1^. MPs consist of plastic microbeads released directly into the environment (primary sources) and plastic fragments derived from the degradation of large plastics (secondary sources) due to weathering processes (e.g. UV photodegradation, mechanical abrasion, biodegradation, etc.)^2^. Degradation processes of plastics are extremely slow, and thus MPs potentially persist for a long time periods in the environment, where degradation may last for decades^3,4^. In addition, MPs are easily driven by wind and water currents, gaining the ability of long-distance diffusion and migration to reach different regions, thus widely distributed in aquatic environment such as surface runoff, rivers, lakes and oceans^5,6^. MPs can accumulate in organisms through the food chains and cause many adverse effects on aquatic and terrestrial organisms, including inhibition of growth and development, endocrine disruption, and immunity and neurotransmission dysfunction^7–9^.

With the characteristics of small size and large specific surface area, MPs always act as carriers to accumulate and transport heavy metal pollutant, and may cause the bioaccumulation of contaminations and toxicants in aquatic environments^10,11^. In recent years, the adsorption properties and mechanisms of heavy metals onto MPs have attracted the attention and research of scientists^12–15^. Holmes et al.^12^ investigated the rates and mechanisms of trace metals (Cr^3+^, Co^2+^, Ni^2+^, Cu^2+^, Zn^2+^, Cd^2+^ and Pb^2+^) binding to virgin and beached polyethylene (PE) pellets, and found that plastics represented an important vehicle for the transport of metals in the marine environment. Gao et al.^13^ observed the significant differences in the effects of different plastic types and locations on the adsorption of different MPs for Pb^2+^, Cu^2+^ and Cd^2+^.

Pb^2+^ is one of the most representative heavy metal pollutants owing to its persistence, bioaccumulation and toxicity in the environment^16^. As a highly toxic metal, Pb^2+^ will affect the morphology, physiology and life activities of animals, plants as well as human beings when it accumulates in living organisms^17–20^. The environmental behavior of Pb^2+^ is affected by MPs contaminants. The interaction between MPs and Pb^2+^ under coexistence conditions not only increases the toxicity of MPs themselves, but also expands the contamination range of Pb^2+^ through the diffusion ability of MPs. Therefore, it is necessary to study the Pb^2+^ adsorption process and mechanism on MPs. Previous studies have showed that MPs can adsorb Pb^2+^ in aquatic environment, and the interaction between MPs and Pb^2+^ is affected by different environmental conditions such as dissolved organic matter (DOM), pH value, ionic strength, temperature and other conditions^13,21–24^. Zou et al.^21^ found that pH can significantly affect the adsorption of Pb^2+^ on chlorinated polyethylene (CPE), polyvinyl chloride (PVC) and polyethylene (PE), but ionic strength exerted a relatively slight effect on this process. Ahechti et al.^23^ found that the adsorption capacity of polyethylene (PE) and polypropylene (PP) for Pb^2+^ was affected by the physicochemical conditions of the aquatic environment (exposure time, pH and salinity). Godoy et al.^24^ investigated the adsorption of Pb^2+^ by five different types of MPs in Milli-Q water and natural waters, and found that an enhancement of metal adsorption in waters with high concentration of DOM. HA, a representative DOM and widely exists in the aqueous environment, is an important natural ligands in regulating the speciation, bioavailability and ultimate fate of trace metal element in the environment^25,26^. HA contains a large number of oxygen-containing functional groups such as carboxyl (–COOH) and hydroxyl groups (–OH), which will interact with Pb^2+^^27^. Our previous study has shown that HA can be adsorbed on PSMPs in the aquatic environment through hydrophobic interaction and π−π electron donor acceptor interaction^28^. Li et al.^29^ found that HA promoted the adsorption of Cd^2+^ on polyvinyl chloride (PVC) and polystyrene (PS) MPs. In addition, it has also been shown that HA increased the adsorption amount of Pb^2+^ on other carbon materials such as activated charcoal particles^30^. These studies suggested that HA could affect the adsorption performance of Pb^2+^ on MPs. However, there were few studies on this relevant mechanism, and more in-depth studies were needed.

In this study, PSMPs were used as model MPs^31^ to study the interaction of Pb^2+^ with PSMPs and the effects of HA on the adsorption process. Adsorption kinetic and isotherm were conducted at different condition (HA concentration, pH value and ionic strength) to research the adsorption characteristic of Pb^2+^ adsorption. The surface morphology of PSMPs before and after adsorption was analyzed using SEM–EDS, and FT-IR as well as XPS were used to study the binding mechanism of PSMPs with Pb^2+^ and HA. In addition, the effect of HA on the adsorption of PSMPs for Pb^2+^ was investigated using fluorescence quenching analysis of HA and site energy distribution theory. The results helped to further understand the characteristics and mechanism of Pb^2+^ adsorption onto MPs, and provide more information for the evaluation of environmental behavior and toxicological effects of MPs in aquatic environments.

## Materials and methods

### Materials and chemicals

Polystyrene microplastics (PSMPs) and Aldrich Humic Acid (HA, sodium salt) were purchased from Sigma-Aldrich (St. Louis, MO, USA). The characteristics of PSMPs were reported in our previous study^31^. Lead nitrate (Pb(NO_3_)_2_), sodium nitrate (NaNO_3_), calcium nitrate (Ca(NO_3_)_2_), nitric acid (HNO_3_) and sodium hydroxide (NaOH) were obtained from Guangzhou Chemical Reagent Co., LTD. (Guangzhou, China). All chemicals were of A.R. grade.

### Sample preparation

The PSMPs were prepared into 50.0 mg/L suspension with ultrapure water (18.2 MΩ). HA solution, obtained by dissolving HA sodium salt in 0.10 mol/L NaOH solution and stirred overnight at 27.0 ± 0.1 ℃, was adjusted to pH 7.0 and then filtered through 0.45-μm cellulose acetate filter paper (Millipore, Billerica, MA, USA). The filtrate was dialyzed with a dialysis membrane (500 D) and finally stored at ~ 4.0 ℃ in the dark. The relevant characterization of HA was shown in the Supplementary data. Pb^2+^ stock solution (500 mg/L) was obtained by dissolving a quantity of Pb(NO_3_)_2_ in distilled water. All the solution pH was adjusted using 0.10 mol/L HNO_3_ or 0.10 mol/L NaOH and measured by an Orion pH/ISE meter (Model 710 A, Thermo Fisher Scientific). The ionic strength was adjusted by adding NaNO_3_ or Ca(NO_3_)_2_ solution, respectively.

### Adsorption experiments

Batch adsorption experiments were employed as described previously with minor modifications^28^. The adsorption kinetic experiments of Pb^2+^ uptake on PSMPs were carried out by adding PSMPs suspension, Pb^2+^ stock solution and HA solution into 500 mL conical flask. The initial Pb^2+^ concentration was 5.00 mg/L and the HA concentrations were 0.00, 1.00, 2.50, 5.00 mg·C/L, respectively. Experimental HA concentrations were determined based on HA concentrations in natural waters ranging from 1 to 10 mg/L^32^. The suspensions were equilibrated on a reciprocating shaker (Shanghai Tensuc Ltd., China) at 27.0 ± 0.1 °C (room temperature) in the dark and sampled at different time within 0–4.0 h. For the adsorption isotherm experiment, the initial Pb^2+^ concentration was 0.50–15.0 mg/L, and the equilibrium time was set at 4.0 h based on preliminary kinetic experiments results. The experiment pH value was adjusted to 3.0 and 6.0 using 0.10 mol/L HNO_3_ or 0.10 mol/L NaOH, and the ionic strength was set to 0.01, 1.00 and 10.0 mmol/L for NaNO_3_, and 0.03, 0.33 and 3.33 mmol/L for Ca(NO_3_)_2_. Pb^2+^ was the predominant form present in the solution^33^. After the experiment, the suspensions were collected and filtered with a 0.45-μm filter membrane, and part of the filtrate is used for Pb^2+^ concentration determination with AAS (Z-2000, Hitachi, Japan), while the other part is used for HA fluorescence detection (RF-5301PC, Shimadzu, Japan). The adsorption amounts of Pb^2+^ adsorbed on PSMPs were calculated from the differences between the initial and final Pb^2+^ concentrations in solutions; mass losses for control samples were negligible (< 1%).

The adsorption process of Pb^2+^ on HA was described in Supplementary data.

The experiments for each condition were performed in triplicate and took the average.

### Analytical method

The zeta potential of PSMPs at different background ionic conditions (Na^+^ or Ca^2+^) were analyzed by Zeta potential analyzer (BI-PALS, Brookhaven, American) at the range of pH 2.0–10.0. The elemental analysis (C, H, N, O, S) of HA was characterized using Elemental analyzer (Vario EL cube, Elementar, Germany). The concentration of HA was measured by TOC analyzer (TOC-L CPH, Shimadzu, Japan). The determation of functional groups (carboxyl and hydroxyl) of HA were performed following the method of Ma et al.^34^. Fluorescence excitation (Ex)-emission (Em) matrix (EEM) spectra of HA was measured at Em wavelength 350–600 nm and Ex wavelength 220–550 nm using a fluorescence spectrometer (RF-5301PC, Shimadzu, Japan). The increments of Em and Ex wavelengths were 1 and 5 nm, and slit bandwidths were settled at 5 nm. The surface morphology and elemental composition of PSMPs were determined using the scanning electron microscope with SEM–EDS (Hitachi U8010, Hitachi, Japan). The functional groups of PSMPs before and after adsorption were characterized using FT-IR (PerkinElmer frontier, American). The FT-IR spectrum was obtained in the wavelength of 400–4000 cm^−1^ with a 1 cm^−1^ resolution. XPS (Thermo Scientific K-Alpha, American) was used to characterize the surface elemental compositions, and the measurement was conducted by using an Al Kα X-ray source at pass energy of 50 eV (for highresolution spectra) and 150 eV (for survey spectra).

Details of the data analysis for Pb^2+^ adsorption onto PSMPs are given in the Supplementary data, and data analysis was performed with Origin Pro 9.0 and MATLAB 2021b for windows.

### Consent to participate

All authors have given consent to their contribution.

### Consent for publication

All authors have agreed with the content and all have given explicit consent to publish.

## Results and discussion

### HA-dependent adsorption of Pb^2+^ onto PSMPs

#### Effect of different HA concentration on Pb^2+^ adsorption onto PSMPs

The adsorption kinetic of Pb^2+^ onto PSMPs at different initial HA concentrations were investigated. As shown in Supplementary Figs. S1 and S2, the adsorption kinetics of Pb^2+^ onto PSMPs with HA were similar to those without HA under different conditions. The adsorption amount of Pb^2+^ was very quick initially and almost completed in the first 60 min, then the *Q*_*t*_ increased quite slowly in 60 ~ 240 min as the contact time increased, and finally reached equilibrium^14^. Consequently, a contact time of 240 min was selected for the following experiments, which was sufficient for the adsorption of Pb^2+^. To further clarify the adsorption kinetics of Pb^2+^ onto PSMPs, pseudo-first-order and pseudo-second-order kinetic models were utilized to fit the experiment data, with the specific fitted parameters shown in Supplementary Tables S1 and S2. The higher *R*^*2*^ values of pseudo-second-order model showed that this model fitted the experimental data better than pseudo-second-order kinetic model. The results also indicated that the chemical adsorption might be the rate-limiting step of Pb^2+^ adsorption mechanism^6,35^. The presence of HA increased the adsorption amount of Pb^2+^ on PSMPs, and the equilibrium absorption capacity *Q*_*e*_ increased with the increase of HA concentration.

**Figure 1:**
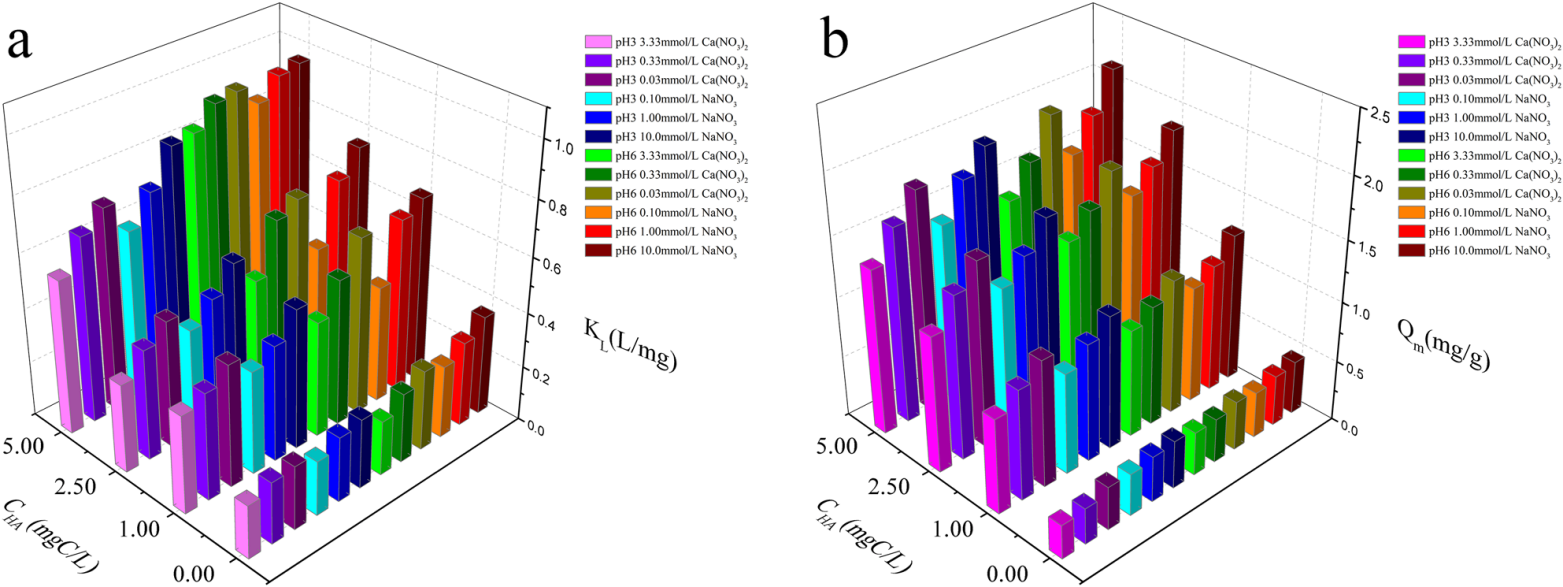
Comparison of Langmuir model parameters *K*_*L*_ and *Q*_*m*_ under different conditions.

The adsorption isotherm curves of Pb^2+^ onto PSMPs in the absence and presence of HA were shown in Supplementary Figs. S3 and S4. In order to analyze adsorption characteristic, the Langmuir and Freundlich isotherm models were used to fit the adsorption data, respectively, and the fitting parameters were listed in Supplementary Tables S3 and S4. The higher *R*^*2*^ values of Langmuir model than Freundlich model suggested that Langmuir model could be better employed for characterizing equilibrium adsorption of Pb^2+^ on PSMPs with/without addition of HA, indicating that the chemisorption and monolayer adsorption played a significant role in the Pb^2+^ adsorption^15,36^. As showed in Fig. 1, *K*_*L*_ and *Q*_*m*_ increased accordingly with the increase of HA concentration, indicating that the existence of HA promoted the adsorption affinity and capacity of PSMPs for Pb^2+^. For example, the adsorption amount of Pb^2+^ on PSMPs increased from 0.443 to 2.13 mg/g when the HA concentrations were 0.00–5.00 mg·C/L, with the condition of pH 6.0 and 0.10 mmol/L NaNO_3_. The results were similar to other heavy metal adsorption in previous studies^29,37^. Li et al.^29^ found that the HA affected the adsorption of Cd^2+^ on PS and PVC MPs. Yang et al.^37^ demonstrated that the presence of HA was beneficial to the adsorption of few-layer reduced graphene oxide (FRGO) and few-layer graphene oxide (FGO) for Cu^2+^.

The promotion effect of HA on the adsorption of Pb^2+^ to PSMPs was presumed to be because HA played an indirect role as a bridge in the adsorption process. In order to study the specific influence process of HA, the adsorption of HA on PSMPs and the adsorption of Pb^2+^ on HA were discussed below. Our previously reported works^28^ had researched the interaction between PSMPs and HA, and it showed that the adsorption of HA on PSMPs conformed to pseudo-second-order kinetic model and Freundlich model. HA adsorbed on PSMPs surface through hydrophobic and π−π interaction (aromatic structure). The adsorption experiments of Pb^2+^ on HA were conducted and the adsorption kinetic and isotherm curves were shown in Supplementary Figs. S5 and S6, as well as the fitting parameters were shown in Supplementary Tables S5 and S6. The pseudo-second-order kinetic model and Langmuir model could be used to describe the adsorption characteristics of Pb^2+^ on HA well. Researches had shown that Pb^2+^ combined with carboxyl and hydroxyl groups in HA molecule to form stable compounds through complexation, ion exchange and electrostatic interaction^38,39^. The results of elemental analysis and group determination of HA also showed that HA had hydroxyl and carboxyl groups (Supplementary Table S7), which was similar to others works^40^.

Comparing the adsorption kinetic results (Supplementary Figs. S1, S2 and S5), the adsorption equilibrium time of Pb^2+^ on HA was shorter than that on PSMPs, illustrating that Pb^2+^ preferentially adsorbed on HA to achieve adsorption equilibrium in PSMPs-HA-Pb^2+^ system. Then HA-Pb^2+^ complex adsorbed onto PSMPs through the interaction between HA and PSMPs, leading to the indirect adsorption of Pb^2+^ onto PSMPs. The higher HA concentration, the more Pb^2+^ indirectly adsorbed. In addition, free Pb^2+^ also adsorbed directly onto PSMPs due to electrostatic interaction, until the concentration of Pb^2+^ in the two phases reached equilibrium.

#### Effect of Ionic strength on Pb^2+^ adsorption onto PSMPs in the presence of HA

Ionic strength played an important role in Pb^2+^ adsorption onto PSMPs in the presence of HA. The *Q*_*e*_ and *V*_*0*_ of adsorption kinetic results (Supplementary Tables S1 and S2) as well as *K*_*L*_ and *Q*_*m*_ of Langmuir model parameters (Fig. 1), decreased with the increase of ionic strength, showing that the higher ionic strength inhibit the adsorption of Pb^2+^ onto PSMPs. For instence, at pH 6.0 and 5.00 mg·C/L HA, the *Q*_*m*_ of Pb^2+^ decreased from 2.13 to 1.57 mg/g, and from 1.97 to 1.42 mg/g as the concentration of Na^+^ and Ca^2+^ increased, showing that the presence of background ions were not favorable for Pb^2+^ adsorption. Similar results were found in previous studies^22,23,33,41^.

The pH_pzc_ of PSMPs in different ionic strength condition was shown in Supplementary Fig. S7. With increasing ionic strength, the pH_pzc_ of PSMPs increased, which was because the charge screening effect by positively charged background ions (Na^+^ or Ca^2+^)^42^. What’s more, the effect of Ca^2+^ on the pH_pzc_ of PSMPs was greater than that of Na^+^ under the same ionic strength condition, as the charge screening effect of divalent positive ions was stronger than that of monovalent positive ions. When the ionic strength increased, the surface negative charge of PSMPs reduced, which resulted in the weakening of electrostatic interaction between PSMPs and Pb^2+^. In addition, according to the DLVO theory, increasing the ionic strength of solution compressed the electric double layer and reduced the electrostatic repulsion, resulting in an increase in the aggregation of PSMPs and a decrease in the effective adsorption sites^2,31^. Competitive adsorption was another reasons for the reduction of Pb^2+^ adsorption. Background electrolyte ions (Na^+^ and Ca^2+^) could compete with Pb^2+^ for specific available adsorption sites on PSMPs and HA molecules^43,44^. Although higher ionic strength was conducive to the adsorption of HA on PSMPs^28^, the effect of high ionic strength condition on the adsorption of Pb^2+^ was much stronger than that on the adsorption of HA. Therefore, high ionic strength ultimately inhibited the adsorption of Pb^2+^ on PSMPs.

#### Effect of pH on Pb^2+^ adsorption onto PSMPs in the presence of HA

The surface charge of the adsorbent, the structure of HA and the ionic species of metals were influenced by the solution pH value, so that the interaction between different substances during adsorption is related to the pH conditions^37^. The adsorption of Pb^2+^ on PSMPs with or without HA at different pH value was studied. As shown in Fig. 1, the *Q*_*m*_ of Pb^2+^ adsorption on PSMPs increased with the increasing solution pH value regardless of the presence of HA. At the condition of 0.10 mmol/L NaNO_3_ and 5.00 mg·C/L HA, the *Q*_*e*_ of Pb^2+^ increased from 1.94 to 2.13 mg/g when pH value ranged from 3.0 to 6.0, indicating that higher pH values was beneficial for the adsorption of Pb^2+^ on PSMPs.

The effect of pH on adsorption is related to the surface charge of PSMPs. The zeta potential of PSMPs gradually decreased with the increase of pH in the range of pH 2.0–10.0 (Supplementary Fig. S7). PSMPs was negatively charged and readily attracted positively charged Pb^2+^ through electrostatic interaction under experimental pH conditions (pH 3.0 and pH 6.0)^15^. The negative charges of PSMPs increased with increasing pH, which led to a corresponding increase in electrostatic interactions between PSMPs and Pb^2+^, and enhanced the adsorption capacity of PSMPs^6^. The decrease in pH also causes competitive adsorption. The large amounts of hydrated hydrogen ions H_3_O^+^ present in solution at low pH conditions competed with Pb^2+^ for adsorption sites on the surface of PSMPs and inhibited the adsorption of Pb^2+^^45,46^. Similar trends were reported for the adsorption of metal ions on other MPs as well as some kind of nanomaterials^14,36,43^.

The addition of HA further increased the role of high pH in promoting the adsorption of Pb^2+^ onto PSMPs. In the presence of HA, the solution pH not only affected the adsorption capacity of Pb^2+^ on PSMPs, but also affected the binding characteristics of HA and Pb^2+^. The adsorption of HA onto PSMPs was little affected by pH, but the adsorption of Pb^2+^ on HA increased with the increase of pH value. The molecular structure of HA was affected by the pH value of solution, i.e., the stretched linear HA structure gradually curled and became a compacted form as the pH decreased^47^. Therefore, HA exposed more functional groups under high pH conditions than low pH conditions, which enhancing its binding with Pb^2+^.

### Different spectroscopic analysis in adsorption process

#### Spectral characterization of PSMPs before and after adsorption

**Figure 2:**
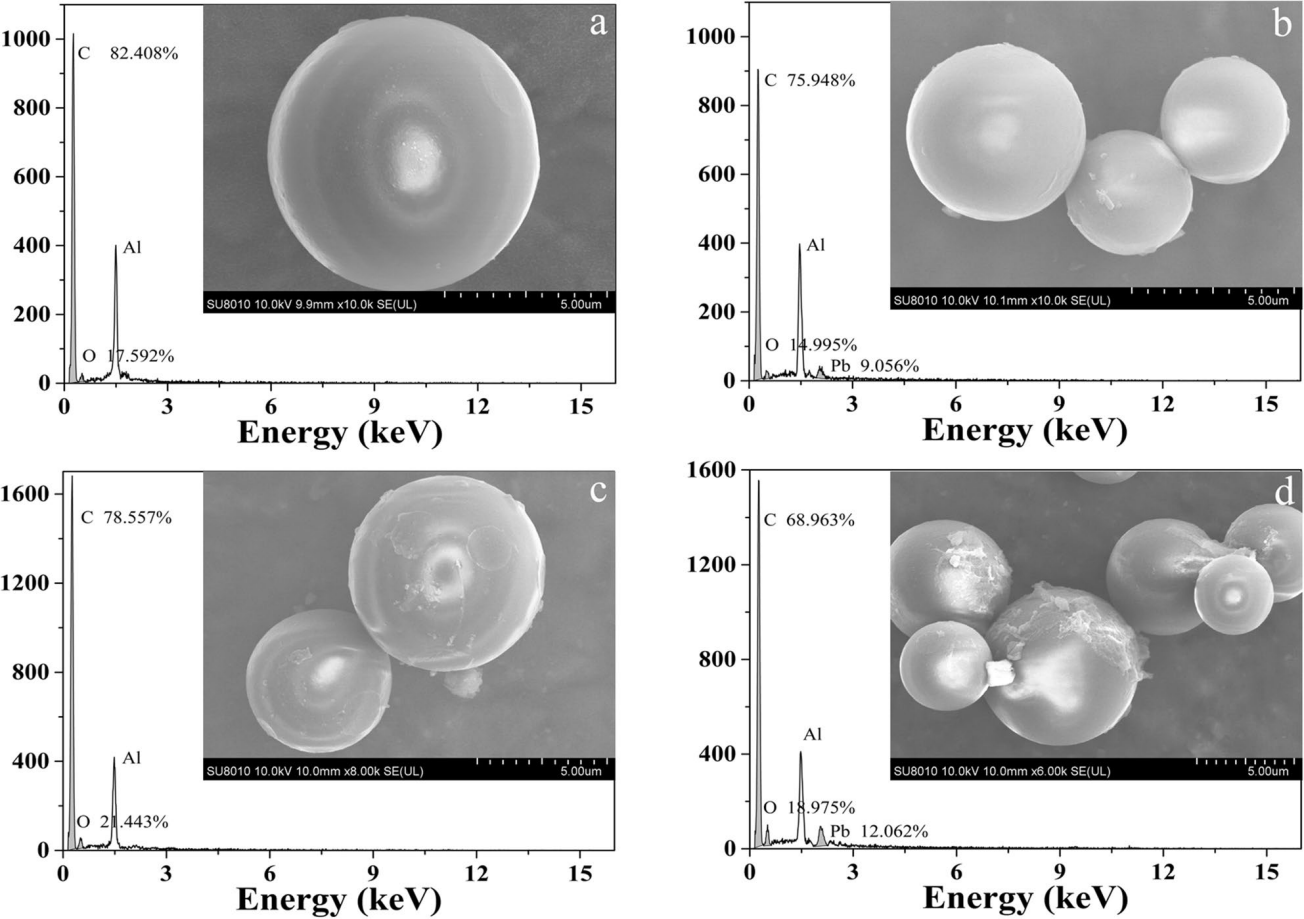
SEM–EDS spectra of PSMPs surface (**a**) virgin PSMPs; (**b**) PSMPs after Pb^2+^ adsorption; (**c**) PSMPs after HA adsorption; (**d**) PSMPs after HA and Pb^2+^ adsorption.

The morphology and microstructure of PSMPs before and after Pb^2+^ adsorption were characterized using SEM–EDS techniques, and the results were shown in Fig. 2. In the absence of HA, the virgin PSMPs particles had globular features and the surface of PSMPs was smooth (Fig. 2a). Slight unevenness of PSMPs was observed in the presence of HA (Fig. 2c), indicating a small amount of HA molecules distributed on the surface of PSMPs. After the adsorption of Pb^2+^, the surface of PSMPs remained relatively smooth when the HA was absence (Fig. 2b), while the surface inhomogeneity of PSMPs increased when HA was presence (Fig. 2d), showing that more HA distributed on the surface of PSMPs. The results of EDS analysis showed that the adsorption of Pb^2+^ onto PSMPs was significantly increased when the HA was presence. The above results showed that, when HA was present, Pb^2+^ could be adsorbed directly onto PSMPs, and also indirectly onto PSMPs by HA.

**Figure 3:**
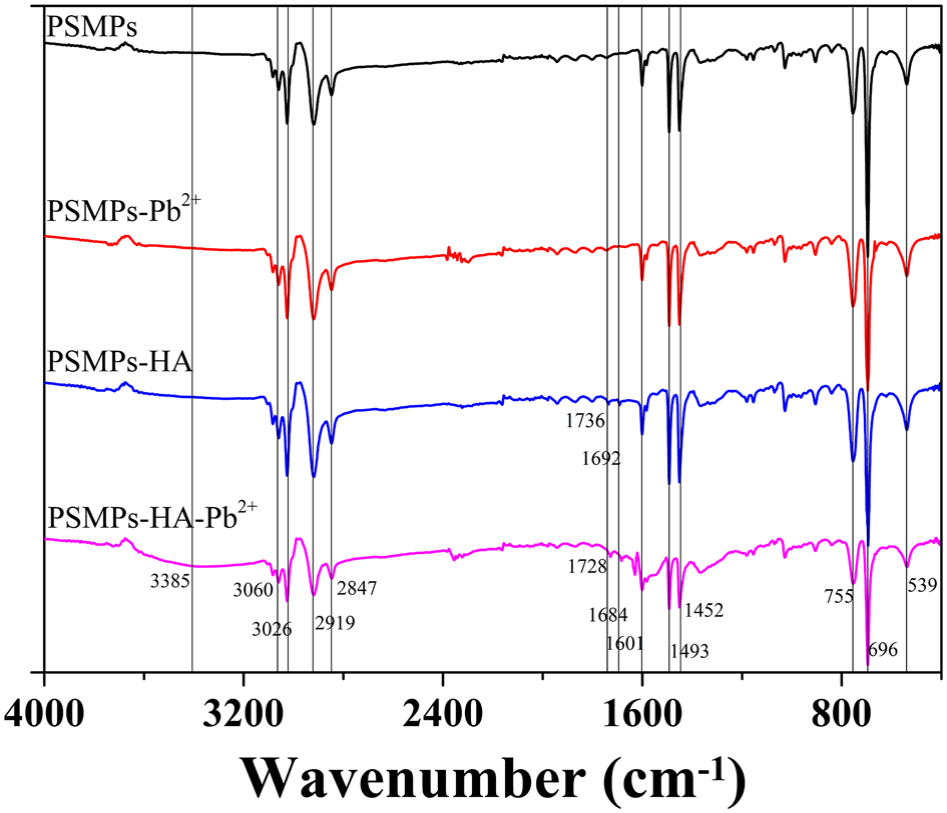
FT-IR spectra of PSMPs before and after HA/Pb^2+^ adsorption.

To explore the structural variation caused by the interaction between Pb^2+^, HA and PSMPs, FT-IR spectra was used to determine the changes in functional groups before and after adsorption, and the results were shown in Fig. 3. Obvious characteristic peaks of aromatic substances were observed in virgin PSMPs particle. The prominent peak appeared at 696, 755, 1452, 1493 and 2840–3100 cm^−1^ were associated with the stretching, deformation and bending vibrations of aromatic ring and aliphatic C–H bonds in PSMPs^48^. The spectra of PSMPs before and after Pb^2+^ adsorption showed almost no change, while after the adsorption of HA, new peaks were observed at 1692 and 1736 cm^−1^ in PSMPs due to the adsorption of HA on PSMPs, which originated from the C=O bond of carboxyl group in HA. After PSMPs adsorbed HA and Pb^2+^, the increased intensity of the peaks at 1692 and 1736 cm^−1^ and the new peak at 3385 cm^−1^ (caused by the O–H stretching vibrations) indicated that more HA was adsorbed onto PSMPs. Moreover, the spectrum of the PSMPs-HA-Pb^2+^ system had a shift of about 8 cm^−1^ in the C=O intensity (1692 and 1736 cm^−1^) compared with that of PSMPs-HA system, confirming the complexation of HA with Pb^2+^, and indirectly proving the increase adsorption of Pb^2+^ onto PSMPs by HA^49^.

**Figure 4:**
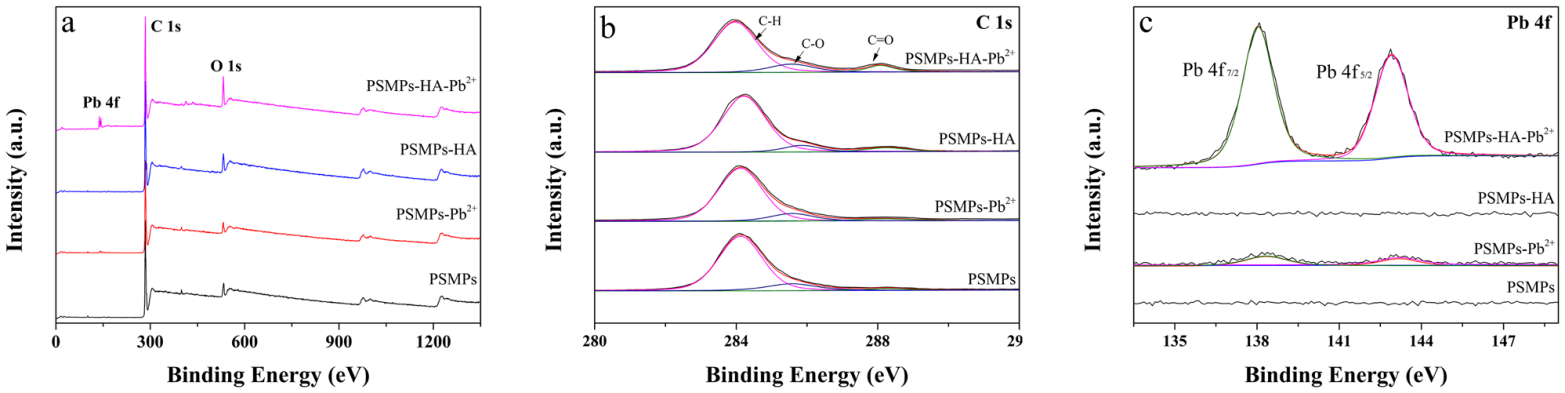
XPS spectra of PSMPs before and after HA/Pb^2+^ adsorption (**a**) survey spectra; (**b**) C 1 s; (**c**) Pb 4f.

Figure 4 showed the XPS spectra of the PSMPs before and after adsorption. From survey spectra (Fig. 4a), the virgin PSMPs particles were consisted of carbon and trace amounts of oxygen. The oxygen atoms contained in the PSMPs may be caused by oxidation during processing or transportation^50^. The C1s spectra of PSMPs (Fig. 4b) showed little difference before and after Pb^2+^ adsorption, while the intensities of C–O (peak at 285.58 eV) and C=O (peak at 288.08 eV) increased after HA adsorption, indicating that HA introduced more oxygen-containing functional groups on PSMPs surface. The high resolution Pb 4f spectra (Fig. 4c) showed two distinguishable peaks that located at 138.08 eV (Pb 4f_7/2_) and 142.88 eV (Pb 4f_5/2_) with an energy separation of 4.8 eV, which were ascribed to Pb^2+^ species adsorbed with the participation of particles oxygen-containing functional groups^45^. In addition, the intensity of Pb 4f. peak increased significantly in the presence of HA, indicating that HA promoted the adsorption of Pb^2+^ on PSMPs, which was consistent with the results of SEM–EDS and FT-IR.

#### Fluorescence quenching analysis of HA in adsorption process

The three-dimensional fluorescence excitation-emission matrix (3DEEM) spectra of HA (Supplementary Fig. S8) showed that the texted HA molecules contained two major fluorescence peaks, i.e., peak A (Ex/Em: 275/481 nm) and peak B (Ex/Em: 455/516 nm). Peak A with high intensity was the fluorescence peak of terrestrial humic-like, while peak B with a lower intensity might be related to microbial metabolism^51^. The peak A with higher intensity was selected for fluorescence quenching analysis in this study.

In HA-Pb^2+^ system and PSMPs-HA-Pb^2+^ system, the fluorescence quenching curves of HA under different conditions were shown in Supplementary Figs. S9–S16. The decrease in fluorescence intensity with increasing Pb^2+^ concentrations revealed a chemical reaction between HA and Pb^2+^. It was also observed that the maximum emission wavelength of HA shifted toward the lower wavelength (i.e. blue shift) with increasing Pb^2+^ concentration, suggesting a possible reduction of conjugated bonds in the chain structure, or the occurrence of π−π* transition in the reaction process^49^.

**Figure 5:**
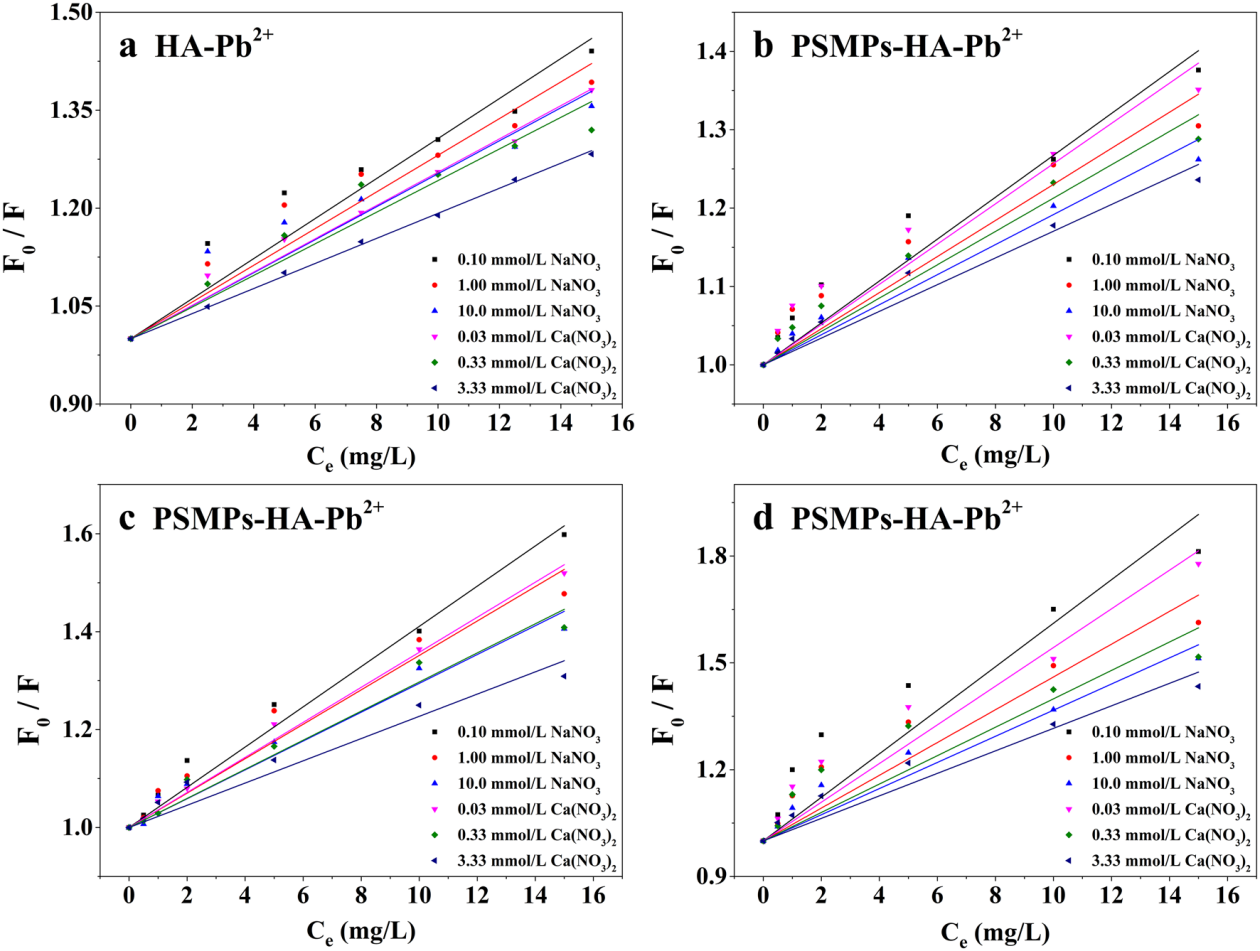
Linear Stern-Volme fitting curves of HA at pH 3.0 (**a**) 5.00 mg·C/L HA; (**b**) 1.00 mg·C/L HA; (**c**) 2.50 mg·C/L HA; (**d** )5.00 mg·C/L HA.

**Figure 6:**
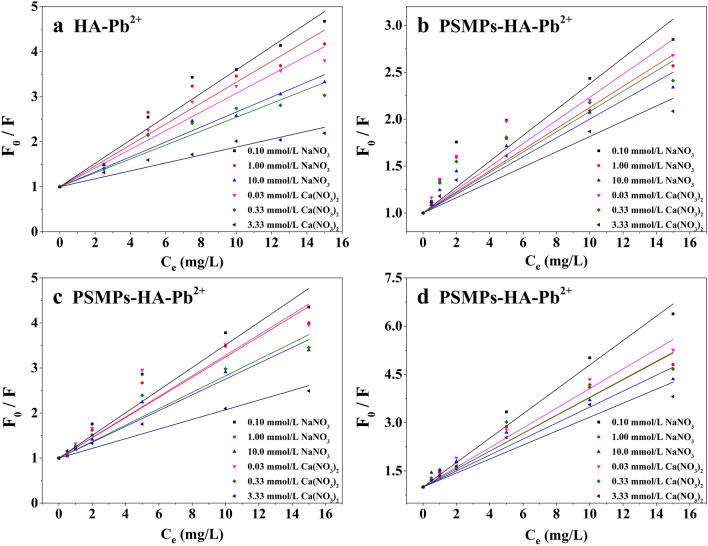
Linear Stern-Volme fitting curves of HA at pH 6.0 (**a**) 5.00 mg·C/L HA; (**b**) 1.00 mg·C/L HA; (**c**) 2.50 mg·C/L HA; (**d**) 5.00 mg·C/L HA.

The linear Stern–Volmer equation was applied to reveal the binding behavior of HA with Pb^2+^, as shown in Figs. 5 and 6, and the model parameter *K*_*SV*_ was shown in Table 1. In the PSMPs-HA-Pb^2+^ system, the fluorescence quenching of HA increased with the increase of pH and the decrease of the ionic strength (Na^+^ and Ca^2+^), which was consistent with the trend in the HA-Pb^2+^ system, demonstrating that PSMPs did not change the binding mode of HA and Pb^2+^. The fluorescence intensity of HA was greatly affected by pH when the ionic strength of the solution was constant. This was because the exposed fluorophore of HA decreased due to the constricted structure of HA molecule at low pH condition, which resulting in the reduction of complexation between HA and Pb^2+^^47^. Under the same pH condition, the effect of ionic strength on the initial fluorescence intensity of HA was mainly caused by the reaction between background ions and fluorophore, which weakened the fluorescence intensity of HA. Therefore, the indirect adsorption of Pb^2+^ on PSMPs through HA decreased at low pH and high ionic strength condition. Compared to the HA-Pb^2+^ system, the parameter *K*_*SV*_ value were higher for the PSMPs-HA-Pb^2+^ system at the same HA concentration (5.00 mg·C/L), indicating that more HA was adsorbed on the surface of PSMPs and bounded to Pb^2+^, and the indirect adsorption of Pb^2+^ (in the form of HA-Pb^2+^ compounds) on PSMPs increased^22^. In PSMPs-HA-Pb^2+^ system, the *K*_*SV*_ value decreased with the decrease of HA concentration, which was attributed to the weak initial fluorescence intensity of HA at lower concentrations and the small change in fluorescence quenching when combined with different concentrations of Pb^2+^.

**Table 1:** *K*_*SV*_ value of fluorescence quenching of HA in different systems.

system	pH	CHA (mg·C/L)	0.100^a^ (mmol/L)	1.00^a^ (mmol/L)	10.0^a^ (mmol/L)	0.0300^b^ (mmol/L)	0.330^b^ (mmol/L)	3.33^b^ (mmol/L)
HA-Pb^2+^	3.0	5.00	0.0307	0.0271	0.0253	0.0255	0.0242	0.0192
	6.0	5.00	0.259	0.232	0.166	0.207	0.154	0.0880
PSMPs-HA-Pb^2+^	3.0	1.00	0.0267	0.0230	0.0192	0.0257	0.0213	0.0171
		2.50	0.0411	0.0351	0.0294	0.0358	0.0297	0.0227
		5.00	0.0611	0.046	0.0367	0.0543	0.0399	0.0316
	6.0	1.00	0.138	0.112	0.100	0.123	0.108	0.0815
		2.50	0.251	0.224	0.175	0.228	0.183	0.107
		5.00	0.379	0.279	0.248	0.306	0.278	0.217

^a^NaNO_3_.

^b^Ca(NO_3_)_2_.

### Site energy distribution analysis for adsorption of Pb^2+^ onto PSMPs promoted by HA

The site energy distribution theory (SEDT) provides relevant information about the energies of adsorption sites, such as high-, low- and average energy sites, and the energy distribution heterogeneity, which can further explain the adsorption mechanism^52^. To further exploring the mechanism of Pb^2+^ adsorbed on PSMPs, the site energy distribution on the surface of PSMPs was calculated based on the Langmuir model and the results were shown in Supplementary Figs. S17 and S18. It could be seen that the E^*^ values gradually decreased with the increase of *Q*_*e*_, indicating that the surface energy distribution of PSMPs was heterogeneous, and the amount of high energy sites were limited. In the adsorption process of PSMPs-HA-Pb^2+^ systems, Pb^2+^ or HA-Pb^2+^ were preferentially adsorbed to the high-energy adsorption sites on PSMPs, followed by low-energy adsorption sites^53^. This was consistent with the adsorption of Cr(VI) on engineered silicate nanoparticles^54^.

**Figure 7:**
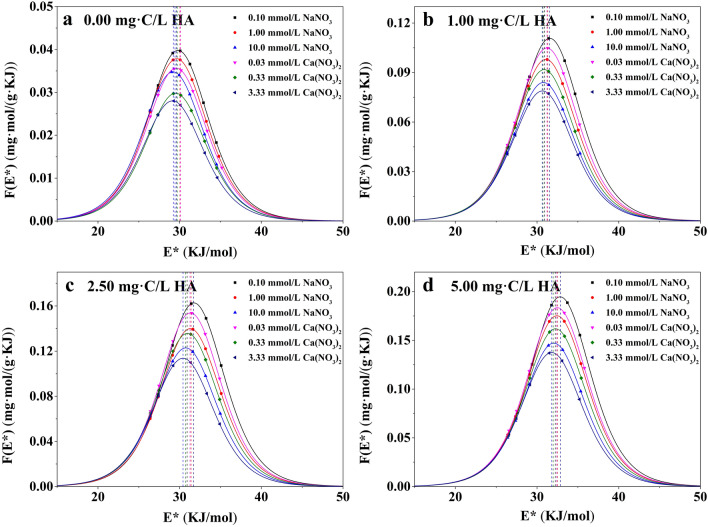
Site energy distribution curves *F*(E^*^) of Pb^2+^ adsorption onto PSMPs at pH 3.0.

**Figure 8:**
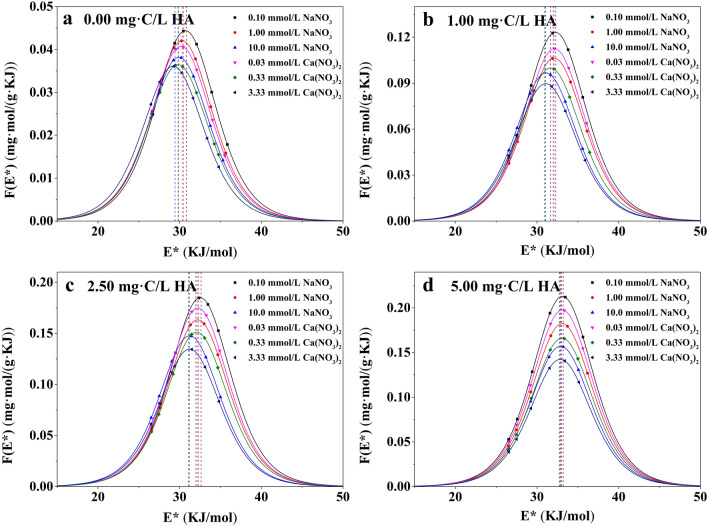
Site energy distribution curves *F*(E^*^) of Pb^2+^ adsorption onto PSMPs at pH 6.0.

Figures 7 and 8 showed that the *F*(E^*^) curves of Pb^2+^ adsorption on PSMPs were all unimodal and quasi-Gaussian^55^. The area under the *F*(E^*^) curve versus adsorption site energy revealed the amount of the available adsorption sites, which could be interpreted as the maximum adsorption capability^56^. The *μ*(E^*^) was higher in the presence of HA than in the absence of HA and increased with increasing HA concentration (Tables 2 and 3), illustrated that the presence of HA could introduce some new adsorption sites, thus enhanced the adsorption site energy on the PSMPs surface and promoted the adsorption of Pb^2+^. In addition, the standard deviation *σ*_e_^*^ which was used to characterize the energy heterogeneity of Pb^2+^ adsorption on PSMPs increased slightly as the increase of HA concentration. This indicated that the adsorption heterogeneity of PSMPs increased after partial HA adsorption on the PSMPs surface, which was consistent with the SEM results.

**Table 2:** Site energy distribution parameters of Pb^2+^ adsorption onto PSMPs at pH 3.0.

C_HA_ (mg·C/L)	Ionic strength (mmol/L)	E_m_^*^(KJ/mol)	F(E_m_^*^) (mg·mol/(g·KJ))	μ(E^*^) (KJ/mol)	σ_e_^*^(KJ/mol)
0.00	0.100^a^	29.8	0.0398	29.8	4.189
	1.00^a^	29.6	0.0379	29.6	4.177
	10.0^a^	29.1	0.0348	29.2	4.174
	0.0300^b^	29.6	0.0356	29.6	4.180
	0.330^b^	29.5	0.0298	29.5	4.176
	3.33^b^	29.1	0.0280	29.1	4.172
1.00	0.100^a^	31.6	0.111	31.6	4.299
	1.00^a^	31.1	0.0980	31.1	4.279
	10.0^a^	30.8	0.0840	30.8	4.258
	0.0300^b^	31.2	0.105	31.2	4.289
	0.330^b^	30.9	0.0918	30.9	4.271
	3.33^b^	30.7	0.0788	30.7	4.255
2.50	0.100^a^	31.7	0.163	31.7	4.441
	1.00^a^	31.3	0.140	31.3	4.401
	10.0^a^	30.8	0.123	30.8	4.388
	0.0300^b^	31.3	0.154	31.3	4.420
	0.330^b^	31.0	0.136	31.0	4.401
	3.33^b^	30.4	0.114	30.4	4.378
5.00	0.100^a^	32.8	0.195	32.8	4.658
	1.00^a^	32.4	0.175	32.4	4.630
	10.0^a^	32.0	0.147	32.0	4.593
	0.0300^b^	32.5	0.183	32.5	4.635
	0.330^b^	32.3	0.162	32.3	4.595
	3.33^b^	31.8	0.137	31.8	4.584

^a^NaNO_3_.

^b^Ca(NO_3_)_2_.

**Table 3:** Site energy distribution parameters of Pb^2+^ adsorption onto PSMPs at pH 6.0.

C_HA_ (mg·C/L)	Ionic strength (mmol/L)	E_m_^*^ (KJ/mol)	F(E_m_^*^) (mg·mol/(g·KJ))	μ(E^*^) (KJ/mol)	σ_e_^*^(KJ/mol)
0.00	0.100^a^	30.7	0.0444	30.7	4.193
	1.00^a^	30.3	0.0420	30.3	4.188
	10.0^a^	29.9	0.0382	29.9	4.183
	0.0300^b^	30.2	0.0407	30.2	4.189
	0.330^b^	29.8	0.0365	29.8	4.186
	3.33^b^	29.2	0.0361	29.2	4.184
1.00	0.100^a^	32.2	0.123	32.2	4.300
	1.00^a^	32.1	0.106	32.1	4.292
	10.0^a^	31.1	0.0967	31.1	4.271
	0.0300^b^	32.1	0.113	32.1	4.293
	0.330^b^	31.7	0.0999	31.7	4.279
	3.33^b^	31.1	0.0897	31.1	4.257
2.50	0.100^a^	32.5	0.185	32.5	4.457
	1.00^a^	32.2	0.163	32.2	4.403
	10.0^a^	31.2	0.148	31.2	4.386
	0.0300^b^	32.2	0.175	32.2	4.422
	0.330^b^	32.1	0.151	32.1	4.401
	3.33^b^	31.2	0.135	31.2	3.386
5.00	0.100^a^	33.1	0.213	33.1	4.662
	1.00^a^	33.0	0.182	33.0	4.631
	10.0^a^	32.9	0.157	32.9	4.596
	0.0300^b^	33.0	0.197	33.0	4.654
	0.330^b^	33.0	0.166	33.0	4.622
	3.33^b^	32.8	0.142	32.8	4.589

^a^NaNO_3_.

^b^Ca(NO_3_)_2_.

The solution pH and ionic strength also affect the site energy distribution of PSMPs. The values of E^*^ and *μ*(E^*^) were higher at pH 6.0 than those at pH 3.0, indicating that there were more adsorption sites for PSMPs at pH 6.0 and resulting in higher adsorption affinity. With increasing ionic strength the site energy decreased, because more binding sites were occupied by salt ions and thus reduced the adsorption capacity of PSMPs. The value of *σ*_e_^*^ for different pH and ionic strength condition were little difference, illustrating that changing the solution pH and ionic strength did not essentially change the surface structure of PSMPs.

## Conclusion

In this article, the adsorption of Pb^2+^ onto PSMPs under different HA concentration was investigated by batch experiments, and the effect of pH and ionic strength condition was also discussed. The adsorption kinetic and isotherm model of Pb^2+^ onto PSMPs conform to the pseudo-second-order kinetics model and Langmuir model, indicating that the process was single layer adsorption and chemical adsorption. Regardless of the presence of HA, the increase of pH and the decrease of ionic strength were favorable for the adsorption of Pb^2+^ on PSMPs surface. HA promoted the adsorption of Pb^2+^ onto PSMPs, and the higher the concentration of HA the greater the adsorption. SEM–EDS, FT-IR and XPS analysis before and after PSMPs HA/Pb^2+^ adsorption suggested that HA adsorbed on PSMPs introduced more functional groups (-COOH and -OH) on the surface of PSMPs and promoted the indirect adsorption capacity of Pb^2+^. The fluorescence quenching of HA in PSMPs-HA-Pb^2+^ systems was stronger than that in HA-Pb^2+^ systems, indicating that more HA was adsorbed on the surface of PSMPs, and the indirect adsorption of Pb^2+^ increased. SEDT analysis showed that the surface energy distribution of PSMPs was heterogeneous, and the addition of HA enhanced the *μ*(E^*^) and *σ*_e_^*^ value of PSMPs, which was favorable for the adsorption of Pb^2+^. The heterogeneity of PSMPs at different pH and ionic strength were similar, indicating that the change of solution conditions did not affect the structure of PSMPs.

These findings were helpful to understand the adsorption process and mechanism of HA affecting on Pb^2+^ adsorption by PSMPs, and could provide a basis for investigating the adsorption of other heavy metals (e.g., Cu^2+^, Zn^2+^, Cr^3+^) onto different MPs (e.g., PE, PVC, PA) in aqueous environment. What’s more, this research was contribute to further evaluating the environmental risks of MPs.

## Supplementary Information

Supplementary Information.

## Data Availability

All data generated or analysed during this study are included in this published article [and its supplementary information files].
