# Supplementary material for: Effects of humic acid on Pb2+ adsorption onto polystyrene microplastics from spectroscopic analysis and site energy distribution analysis
Source: Sci Rep. 2022 May 27;12:8932. doi: 10.1038/s41598-022-12776-3 (PMC9142603; doi:10.1038/s41598-022-12776-3)
Supplement: Supplementary file 1 — Supplementary Information. [file 41598_2022_12776_MOESM1_ESM.pdf]

Supplementary data

Effects of Humic Acid on Pb^{2+} Adsorption onto Polystyrene Microplastics from Spectroscopic Analysis and Site Energy Distribution Analysis

Xiaotian Lu[a], Feng Zeng[a], Shuyin Wei [a], Rui Gao[a], Abliz Abdurahman[a], Hao Wang [a], Weiqian Liang[a*]

*corresponding author

[a]School of Chemistry, Sun Yat-sen University, Guangzhou, 510275, China

E-mail: liangwq3@mail2.sysu.edu.cn

Phone: 020-84114133

This Supplementary data includes a total of 32 pages (including this page) with sections for experimental, references, and 18 figures and 7 tables.

1. Adsorption of Pb^{2+} on HA

The reaction of Pb^{2+} with HA fractions was carried out at two pH values (3.0 and 6.0) and six ionic strength (0.01, 1.00 and 10.0 mmol/L for $NaNO_3$, and 0.03, 0.33 and 3.33mmol/L for $Ca(NO_3)_2$). The adsorption kinetic experiments of Pb^{2+} uptake on HA was carried at 27.0±1.0℃ with an initial concentration of 5.00 mg·C/L HA and 5.00 mg/L Pb^{2+}. The ultrapure water, Pb^{2+} solution and background electrolyte solution were added to a 250 mL beaker to achieve the preset concentration of each component. The pH value of the solution was adjusted to 3.0/6.0 with HNO_3 in advance. PXSJ-216F ion meter (PXSJ-216F, Rex Electric Chemical, China) and Pb ion selective electrode (Pb-ISE) (PPb-1-01, Rex Electric Chemical, China) were used to determine the initial concentration of Pb^{2+} in the solution under stirring conditions. Then HA solution was added to the mixed solution, and the fixed time measurement mode was selected to measure the concentration of Pb^{2+} in the solution at different time within 0 to 60 min. In adsorption isotherm experiment, HA concentration was set as 5.00 mg·C/L, and the initial concentration gradient of Pb^{2+} was 2.50, 5.00, 7.50, 10.0, 12.5 and 15.0 mg/L. The experimental process was similar to adsorption kinetic experiment, and the equilibrium time was set at 60min. The concentration of Pb^{2+} before adsorption and after equilibrium was determined by PXSJ-216F ion meter and Pb-ISE. 5ml solution was taken for filtration, and the filtrate was used for fluorescence spectrometry fluorescence detection (RF-5301PC, Shimadzu, Japan).

2. Data analysis

2.1 Adsorption kinetics

Pseudo-first-order kinetic model and pseudo-second-order kinetic model were used to fit the experiment data, and the equation were generally expressed as

follows[1,2]:

Pseudo-first-order kinetic model:

$$\log（Q_e\text{-}Q_t）=\log Q_e\text{-}\frac{k_1}{2.303}t \qquad （1）$$

Pseudo-second-order kinetic model:

$$\frac{t}{Q_t}=\frac{1}{k_2 * Q_e^2}+\frac{t}{Q_e} \qquad （2）$$

Where t is adsorption time, min; Q_t and Q_e are the adsorption capacity of Pb^{2+} at time t and equilibrium, mg/g; k_1 is the rate constant of the pseudo-first-order kinetic model, 1/min; k_2 is the rate constant of the pseudo-first-order kinetic model, g/(mg·min).

2.2 Adsorption isotherm

Adsorption isotherms of Pb^{2+} on PSMPs were fitted by Langmuir and Freundlich isotherm model, which are described as follow[3,4]:

Langmuir model:

$$\frac{C_e}{Q_e}=\frac{1}{K_L * Q_m}+\frac{C_e}{Q_m} \qquad （3）$$

Freundlich model:

$$\log Q_e=\log K_F-\frac{1}{n}\log C_e \qquad （4）$$

Where C_e is the final concentration of Pb^{2+} at adsorption equilibrium, mg/L; Q_e is the adsorption amount of Pb^{2+} on PSMPs at equilibrium, mg/g; Q_m is the theoretical maximum adsorption capacity of Pb^{2+}, mg/g; K_L is Langmuir adsorption constant, L/mg; K_F is Freundlich adsorption constant, L/g; n is dimensionless empirical constant, which represents the adsorption intensity and nonlinearity of adsorption isotherm.

The Langmuir model assumes that the surface of the adsorbent is uniform and the number of adsorption sites is fixed, as well as the adsorbed molecules form a monolayer on the surface of the adsorbent. The Langmuir adsorption constant K_L related to adsorption capacity, and the larger the value of K_L, the stronger the adsorption capacity of the adsorbent. The Freundlich model describes the adsorption process of chemical (monolayer) adsorption and physical (multilayer) adsorption on a heterogeneous surface. The correlation constant K_F represents the adsorption capacity of the adsorbent, and the value of *n* represents the heterogeneity of adsorbent, as well as relates to intensity of adsorption[5].

2.3 Site energy distribution theory

The site energy distribution theory (SEDT) is a theoretical method to study the surface energy inhomogeneity and heterogeneous adsorption mechanism of adsorbents[6,7]. According to the adsorption theory of heterogeneous surfaces, the site energy distribution is determined by the integral equation, which represents the adsorption isothermal general formula of heterogeneous surfaces:

$$Q_e(C_e) = \int_0^{+\infty} Q_h(E, C_e) * F(E) dE \qquad (5)$$

Where C_e is the equilibrium concentration of adsorbate in liquid phase, $Q_e(C_e)$ is the total adsorption of solute to the adsorbent, $Q_h(E, C_e)$ is the isotherm over local sorption sites with adsorption energy *E*, and *F(E)* is the site energy frequency distribution over a range of sites with homogeneous energies. Adsorption energy *E* refers to the difference of adsorption energies between the solute and solvent for a given sorption site[8].

The relationship between E^* and Ce can be obtained by "approximate condensation method" as follows:

$$C_e = C_S * \exp(-\frac{E - E_S}{RT}) = C_S * \exp(-\frac{E^*}{RT}) \qquad (6)$$

Where C_e is the concentration of Pb^{2+} in the solution at equilibrium, mol/L; C_s is the maximum solubility of Pb^{2+} in the solution, g/L; R is the universal gas constant 8.314 J/(mol·K); T is the absolute temperature, K; E and Es are the adsorption energies for Ce and Cs, kJ/mol.

According to the relationship between Ce and E^*, the adsorption isotherm model can be described as a function $Q_e(E^*)$ related to E^*, and the approximate site energy distribution function $F(E^*)$ can be obtained by differentiating $Q_e(E^*)$

$$F(E^*) = \frac{Q_m K_L C_S}{RT} \exp(\frac{-E^*}{RT}) \left[1 + K_L C_S \exp(\frac{-E^*}{RT}) \right]^{-2} \qquad (7)$$

The formulas of mean $\mu(E^*)$ and standard deviation (σ_e^*) of adsorption site energy were constructed by mathematical expectation and standard deviation:

$$\mu(E^*) = \frac{\int_0^{+\infty} E^* * F(E^*) dE^*}{\int_0^{+\infty} F(E^*) dE^*} \qquad (8)$$

$$\mu(E^{*2}) = \frac{\int_0^{+\infty} E^{*2} * F(E^*) dE^*}{\int_0^{+\infty} F(E^*) dE^*} \qquad (9)$$

$$\sigma_e^* = \sqrt{\mu(E^{*2}) - \mu(E^*)^2} \qquad (10)$$

The peak value of site energy distribution curve can be expressed as $F(E_m^*)$, and the corresponding E_m^* can be expressed as the following formula:

$$E_m^* = RT \ln(K_L * C_S) \qquad (11)$$

2.4 Fluorescence quenching analysis

Fluorescence quenching is an analytical technique based on the actual molecular contact between the fluorophore and the quenching agent with high sensitivity. The Stern-Volmer equation is used to fit the fluorescence quenching data of HA with the addition of Pb^{2+}[9,10]:

$$F_0/F = 1 + K_{SV}[Q] \qquad (12)$$

Where F_0 and F are the HA fluorescence intensity in the absence and presence of Pb^{2+}, respectively; K_{SV} is the Stern-Volmer quenching constant; [Q] is the concentration of Pb^{2+}, mg/L.

Data analysis was performed with OriginPro 9.0 for windows.

3. References

1. Ho, Y. S. Citation review of Lagergren kinetic rate equation on adsorption reactions. *entometrics* **59**, 171-177 (2004).

2. Ho, Y. S. Review of Second-Order Models for Adsorption Systems. *ChemInform* **131**, 681-689 (2006).

3. Langmuir, I. The Adsorption of Gases on Plane Surfaces of Glass, Mica and Platinum. *Journal of the American Chemical Society* **40**, 1361-1403 (1917).

4. Yang, C. Statistical Mechanical Study on the Freundlich Isotherm Equation. *J Colloid Interface Sci* **208**, 379-387 (1998).

5. Zeng, Z. *et al.* Research on the sustainable efficacy of g-MoS_2 decorated biochar nanocomposites for removing tetracycline hydrochloride from antibiotic-polluted aqueous solution. *ence of The Total Environment* **648**, 206-217 (2018).

6. Carter, M. C., Kilduff, J. E. & Weber, W. J. Site energy distribution analysis of preloaded adsorbents. *Environmental Science & Technology* **29**, 1773-1780 (1995).

7. Huang, L. *et al.* High-resolution insight into the competitive adsorption of heavy metals on natural sediment by site energy distribution. *Chemosphere* **197**, 411 (2018).

8. Yan, B., Niu, C. & Wang, J. Analyses of Levofloxacin Adsorption on Pretreated Barley Straw with Respect to Temperature: Kinetics, π-π Electron-Donor-Acceptor Interaction and Site Energy Distribution. *Environmental Science & Technology* **122**, 128 (2017)

9. Puchalski, M. M., Morra, M. J. & Wandruszka, R. V. Assessment of inner filter effect corrections in fluorimetry. *Fresenius' Journal of Analytical Chemistry* **340**, 341-344 (1991).

10. Ke *et al.* A multi-method analysis of the interaction between humic acids and heavy metal ions. *Journal of Environmental Science & Health Part A Toxic/hazardous Substances & Environmental Engineering* **53**,740-751(2018).

Table S1 Adsorption kinetic model parameters for Pb^{2+} adsorption onto PSMPs at pH 3.0

C_{HA}	Ionic Strength	Pseudo - first- order kinetic model			Pseudo - second- order kinetic model			
(mg·C/L)	(mmol/L)	k_1(1/min)	Q_e(mg/g)	R^2	k_2(g/(mg·min))	Q_e(mg/g)	V_0(mg/(g·min)	R^2
0.00	0.100[a]	0.0668	0.254	0.962	0.337	0.282	0.0268	0.979
	1.00[a]	0.0559	0.225	0.965	0.307	0.251	0.0194	0.979
	10.0[a]	0.0546	0.193	0.968	0.341	0.216	0.016	0.978
	0.0300[b]	0.0592	0.202	0.957	0.375	0.224	0.0189	0.977
	0.330[b]	0.0677	0.163	0.956	0.535	0.181	0.0175	0.98
	3.33[b]	0.0751	0.131	0.903	0.769	0.144	0.016	0.951
1.00	0.100[a]	0.0758	0.753	0.96	0.134	0.83	0.092	0.979
	1.00[a]	0.0877	0.632	0.975	0.183	0.696	0.0883	0.99
	10.0[a]	0.0551	0.49	0.986	0.132	0.552	0.0401	0.976
	0.0300[b]	0.0805	0.677	0.956	0.157	0.746	0.0874	0.983
	0.330[b]	0.0683	0.55	0.989	0.151	0.614	0.0569	0.984
	3.33[b]	0.0441	0.447	0.978	0.111	0.508	0.0287	0.974
2.50	0.100[a]	0.097	1.16	0.952	0.115	1.27	0.184	0.98
	1.00[a]	0.101	0.938	0.975	0.138	1.03	0.148	0.986
	10.0[a]	0.0989	0.807	0.969	0.164	0.885	0.128	0.99
	0.0300[b]	0.0965	1.08	0.966	0.118	1.19	0.166	0.986
	0.330[b]	0.118	0.882	0.977	0.177	0.964	0.164	0.982
	3.33[b]	0.138	0.646	0.925	0.295	0.702	0.145	0.974
5.00	0.100[a]	0.109	1.45	0.943	0.109	1.57	0.269	0.976
	1.00[a]	0.153	1.21	0.953	0.187	1.3	0.316	0.988
	10.0[a]	0.12	0.974	0.976	0.167	1.06	0.188	0.987
	0.0300[b]	0.137	1.34	0.972	0.146	1.45	0.307	0.988
	0.330[b]	0.0912	1.12	0.958	0.107	1.24	0.164	0.986
	3.33[b]	0.105	0.867	0.981	0.156	0.953	0.142	0.992

[a]$NaNO_3$
[b]$Ca(NO_3)_2$

Table S2 Adsorption kinetic model parameters for Pb^{2+} adsorption onto PSMPs at pH 6.0

C_{HA} (mg·C/L)	Ionic Strength (mmol/L)	Pseudo - first- order kinetic model			Pseudo - second- order kinetic model			
		k_1(1/min)	Q_e(mg/g)	R^2	k_2(g/(mg·min))	Q_e(mg/g)	V_0(mg/(g·min)	R^2
	0.100[a]	0.0804	0.274	0.936	0.390	0.302	0.0356	0.964
	1.00[a]	0.0644	0.245	0.954	0.330	0.273	0.0245	0.978
0.00	10.0[a]	0.0673	0.209	0.945	0.419	0.232	0.0225	0.978
	0.0300[b]	0.0735	0.225	0.934	0.423	0.250	0.0264	0.967
	0.330[b]	0.0685	0.201	0.949	0.438	0.223	0.0217	0.973
	3.33[b]	0.0732	0.171	0.952	0.537	0.191	0.0196	0.979
	0.100[a]	0.0749	0.810	0.937	0.128	0.888	0.101	0.970
	1.00[a]	0.0846	0.661	0.937	0.164	0.732	0.0881	0.981
1.00	10.0[a]	0.0591	0.580	0.977	0.124	0.650	0.0522	0.987
	0.0300[b]	0.0730	0.740	0.939	0.132	0.817	0.0877	0.973
	0.330[b]	0.0668	0.622	0.961	0.135	0.693	0.0649	0.984
	3.33[b]	0.0546	0.557	0.984	0.116	0.626	0.0456	0.977
	0.100[a]	0.100	1.29	0.938	0.107	1.41	0.214	0.979
	1.00[a]	0.0882	1.17	0.967	0.101	1.28	0.166	0.986
2.50	10.0[a]	0.0932	0.988	0.966	0.126	1.09	0.148	0.985
	0.0300[b]	0.107	1.22	0.941	0.124	1.33	0.218	0.979
	0.330[b]	0.0791	1.08	0.966	0.0944	1.20	0.135	0.988
	3.33[b]	0.0951	0.862	0.964	0.150	0.943	0.133	0.981
	0.100[a]	0.118	1.62	0.962	0.101	1.76	0.313	0.984
	1.00[a]	0.149	1.35	0.948	0.162	1.46	0.343	0.984
5.00	10.0[a]	0.114	1.09	0.960	0.149	1.18	0.208	0.985
	0.0300[b]	0.145	1.50	0.957	0.137	1.62	0.361	0.982
	0.330[b]	0.129	1.21	0.938	0.148	1.31	0.255	0.980
	3.33[b]	0.116	0.948	0.938	0.175	1.03	0.185	0.969

[a]$NaNO_3$
[b]$Ca(NO_3)_2$

Table S3 Adsorption isotherm model parameters for Pb^{2+} adsorption onto PSMPs at pH 3.0

C_{HA} (mg·C/L)	Ionic Strength (mmol/L)	Langmuir model K_L(L/mg)	Q_m(mg/g)	R^2	Freundlich model K_F(L/g)	n	R^2
0.00	0.100[a]	0.254	0.397	0.993	0.0971	2.21	0.933
	1.00[a]	0.237	0.378	0.990	0.0859	2.11	0.926
	10.0[a]	0.199	0.347	0.988	0.0698	1.99	0.933
	0.0300[b]	0.232	0.356	0.995	0.0793	2.09	0.941
	0.330[b]	0.225	0.297	0.985	0.0658	2.10	0.930
	3.33[b]	0.194	0.280	0.974	0.0550	1.98	0.918
1.00	0.100[a]	0.513	1.11	0.996	0.400	2.83	0.952
	1.00[a]	0.424	0.978	0.993	0.324	2.68	0.965
	10.0[a]	0.376	0.838	0.980	0.255	2.51	0.981
	0.0300[b]	0.446	1.05	0.994	0.351	2.69	0.955
	0.330[b]	0.391	0.916	0.988	0.284	2.54	0.965
	3.33[b]	0.358	0.787	0.985	0.232	2.46	0.987
2.50	0.100[a]	0.552	1.62	0.967	0.670	3.34	0.873
	1.00[a]	0.462	1.40	0.967	0.519	3.04	0.884
	10.0[a]	0.391	1.23	0.990	0.399	2.73	0.936
	0.0300[b]	0.466	1.54	0.982	0.576	3.06	0.924
	0.330[b]	0.405	1.36	0.982	0.466	2.86	0.906
	3.33[b]	0.324	1.13	0.986	0.340	2.57	0.932
5.00	0.100[a]	0.854	1.94	0.985	1.05	4.55	0.919
	1.00[a]	0.727	1.75	0.980	0.886	4.22	0.923
	10.0[a]	0.624	1.47	0.977	0.693	3.87	0.931
	0.0300[b]	0.743	1.83	0.988	0.921	4.09	0.924
	0.330[b]	0.680	1.62	0.990	0.795	4.07	0.926
	3.33[b]	0.565	1.37	0.979	0.613	3.60	0.939

[a] $NaNO_3$

[b] $Ca(NO_3)_2$

Table S4 Adsorption isotherm model parameters for Pb^{2+} adsorption onto PSMPs at pH 6.0

C_{HA} (mg·C/L）	Ionic Strength (mmol/L)	Langmuir model			Freundlich model		
		K_L(L/mg)	Q_m(mg/g)	R^2	K_F(L/g)	n	R^2
0.00	0.100[a]	0.364	0.443	0.998	0.149	2.71	0.961
	1.00[a]	0.310	0.419	0.999	0.128	2.56	0.965
	10.0[a]	0.265	0.381	0.998	0.106	2.42	0.970
	0.0300[b]	0.293	0.406	0.998	0.120	2.49	0.968
	0.330[b]	0.254	0.364	0.995	0.0996	2.42	0.971
	3.33[b]	0.199	0.360	0.993	0.0818	2.16	0.977
1.00	0.100[a]	0.676	1.23	0.991	0.499	3.06	0.966
	1.00[a]	0.635	1.06	0.984	0.412	2.91	0.978
	10.0[a]	0.426	0.965	0.971	0.309	2.52	0.984
	0.0300[b]	0.646	1.12	0.990	0.442	2.97	0.962
	0.330[b]	0.534	0.997	0.979	0.356	2.72	0.982
	3.33[b]	0.423	0.895	0.985	0.285	2.53	0.976
2.50	0.100[a]	0.753	1.85	0.982	0.844	3.57	0.923
	1.00[a]	0.667	1.63	0.987	0.716	3.41	0.962
	10.0[a]	0.450	1.47	0.988	0.530	2.87	0.973
	0.0300[b]	0.670	1.75	0.994	0.772	3.47	0.932
	0.330[b]	0.631	1.51	0.995	0.648	3.36	0.957
	3.33[b]	0.448	1.34	0.990	0.485	2.89	0.972
5.00	0.100[a]	0.958	2.13	0.982	1.03	3.68	0.959
	1.00[a]	0.943	1.82	0.967	0.901	3.76	0.964
	10.0[a]	0.875	1.57	0.984	0.747	3.68	0.957
	0.0300[b]	0.947	1.97	0.985	0.953	3.68	0.949
	0.330[b]	0.936	1.66	0.994	0.811	3.79	0.928
	3.33[b]	0.865	1.42	0.973	0.662	3.53	0.976

[a] $NaNO_3$

[b] $Ca(NO_3)_2$

Table S5 Adsorption kinetic model parameters for Pb^{2+} adsorption on HA

pH	Ionic Strength (mmol/L)	Pseudo - first- order kinetic model			Pseudo - second-order kinetic model			
		k_1(1/min)	Q_e(mg/g·C)	R^2	k_2(10^{-4}·g·C/(mg·min))	Q_e(mg/g·C)	V_0(mg/(g·C·min)	R^2
3.00	0.100[a]	0.345	209	0.966	19.4	229	102	0.991
	1.00[a]	0.436	170	0.973	31.4	184	107	0.993
	10.0[a]	0.447	137	0.972	41.9	147	90.7	0.995
	0.0300[b]	0.407	183	0.982	27.0	198	106	0.984
	0.330[b]	0.470	144	0.953	40.0	155	96.5	0.990
	3.33[b]	0.248	112	0.975	26.6	124	40.7	0.991
6.00	0.100[a]	0.300	353	0.976	10.8	385	160	0.994
	1.00[a]	0.262	305	0.964	11.0	334	123	0.991
	10.0[a]	0.298	251	0.978	13.8	277	106	0.995
	0.0300[b]	0.299	309	0.929	12.6	337	143	0.978
	0.330[b]	0.184	276	0.977	8.22	307	77.7	0.993
	3.33[b]	0.151	220	0.935	9.03	245	54.4	0.974

[a]$NaNO_3$
[b]$Ca(NO_3)_2$

Table S6 Adsorption isotherm model parameters for Pb^{2+} adsorption on HA

pH	Ionic Strength (mmol/L)	Langmuir model			Freundlich model		
		K_L(L/mg)	Q_m(mg/g·C)	R^2	K_F(L/g)	n	R^2
3.00	0.100[a]	0.229	470	0.973	120	2.33	0.904
	1.00[a]	0.217	404	0.971	98.7	2.26	0.901
	10.0[a]	0.206	325	0.985	77.2	2.23	0.971
	0.0300[b]	0.213	447	0.978	107	2.22	0.917
	0.330[b]	0.211	361	0.998	85.8	2.21	0.978
	3.33[b]	0.198	294	0.998	67.2	2.18	0.985
6.00	0.100[a]	0.354	778	0.988	252	2.58	0.946
	1.00[a]	0.258	722	0.993	193	2.30	0.980
	10.0[a]	0.242	601	0.973	155	2.28	0.908
	0.0300[b]	0.297	755	0.985	219	2.39	0.946
	0.330[b]	0.249	638	0.998	168	2.31	0.972
	3.33[b]	0.222	518	0.994	127	2.24	0.975

[a]$NaNO_3$

[b]$Ca(NO_3)_2$

Table S7 Elemental analysis and acid functional group determination of HA

Material	C(%)	H(%)	O(%)	N(%)	S(%)	C/H	O/C	Q_{total} (mol/kg)	Q_{COOH} (mol/kg)	Q_{OH} (mol/kg)
HA	52.63	4.28	42.04	1.17	0.54	1.02	0.59	4.94	3.61	1.33

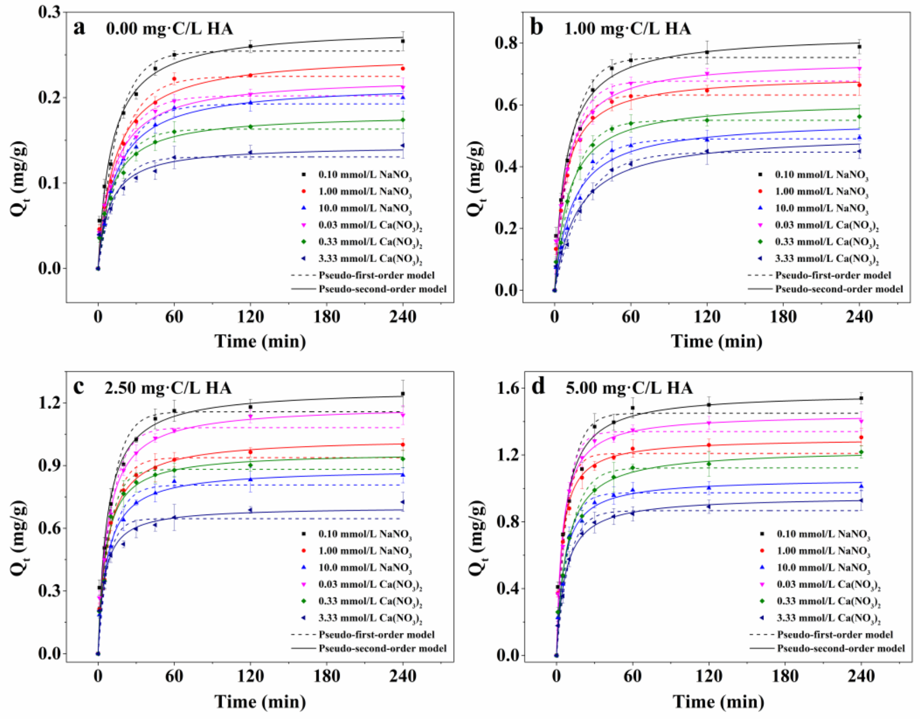

Fig. S1 Adsorption kinetic curves of Pb^{2+} onto PSMPs under different condition at pH 3.0

(mean value ± SD, n=3)

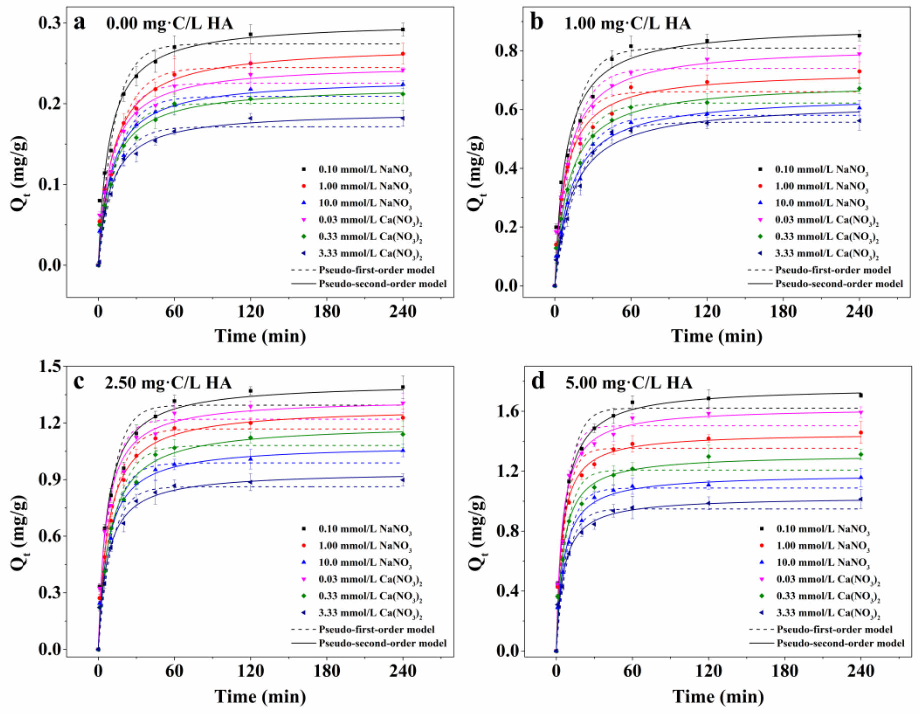

Fig. S2 Adsorption kinetic curves of Pb^{2+} onto PSMPs under different condition at pH 6.0

(mean value ± SD, n=3)

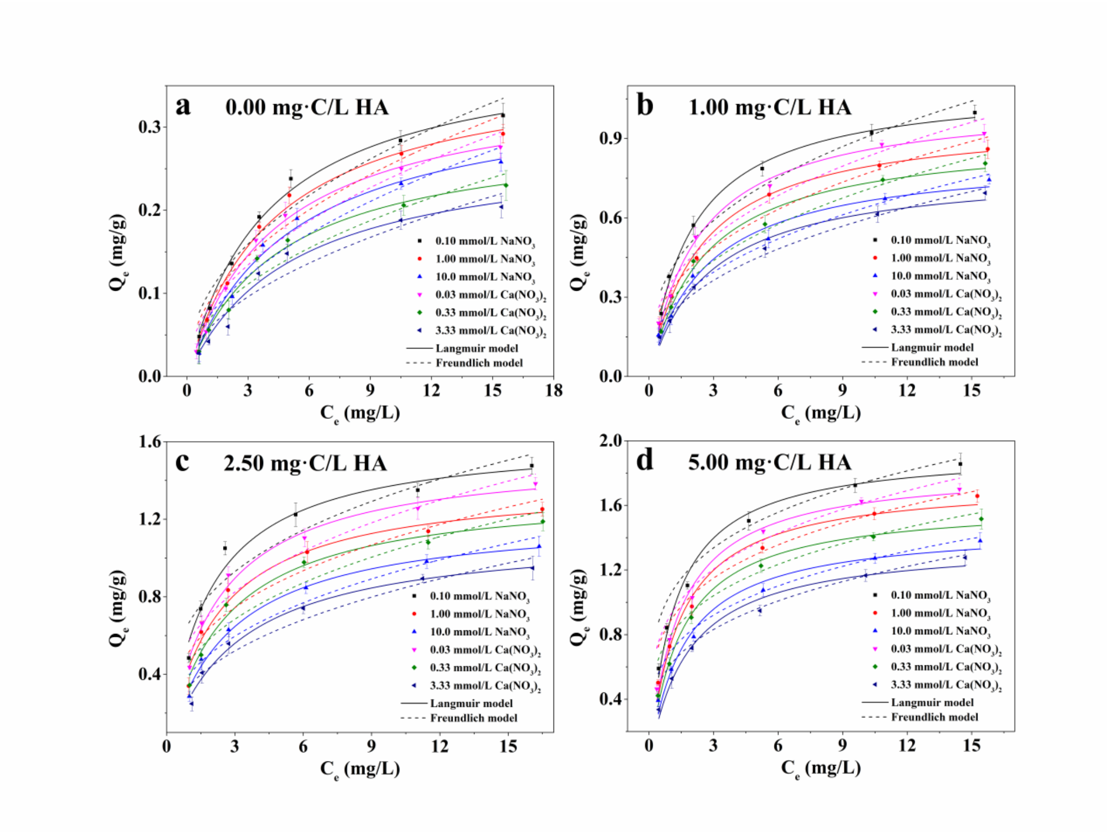

Fig. S3 Adsorption isotherm curves of Pb^{2+} onto PSMPs under different condition at pH 3.0

(mean value ± SD, n=3)

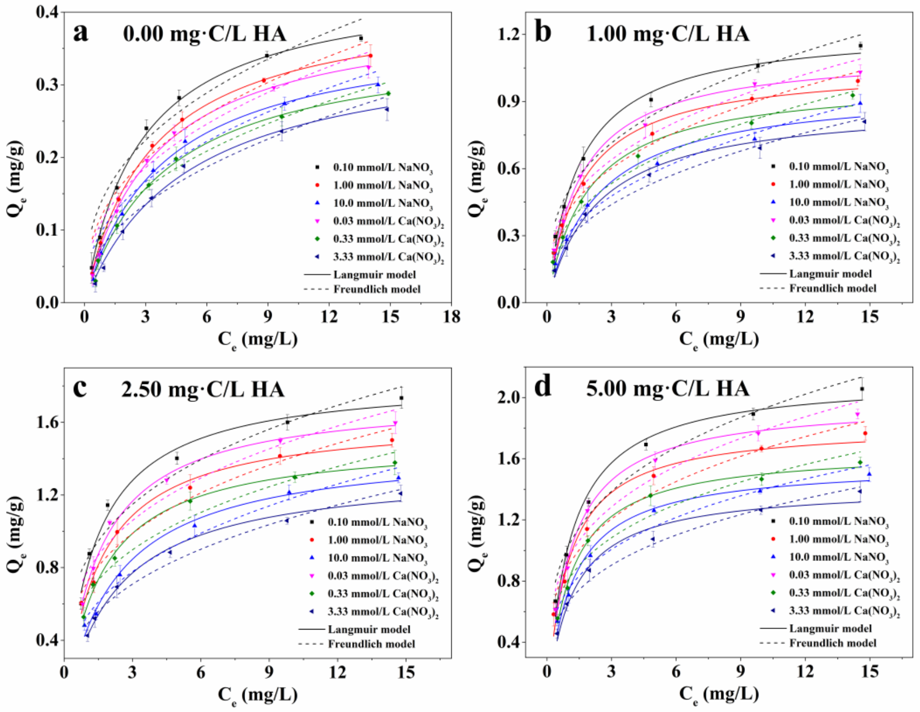

Fig. S4 Adsorption isotherm curves of Pb^{2+} onto PSMPs under different condition at pH 6.0

(mean value ± SD, n=3)

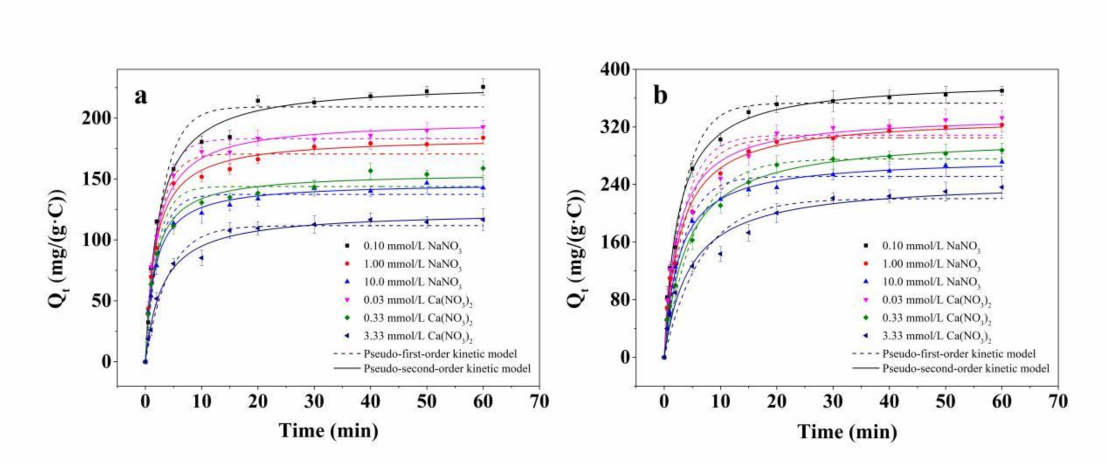

Fig. S5 Adsorption kinetic curves of Pb^{2+} on HA under different condition (a. pH 3.0, b. pH 6.0)

(mean value ± SD, n=3)

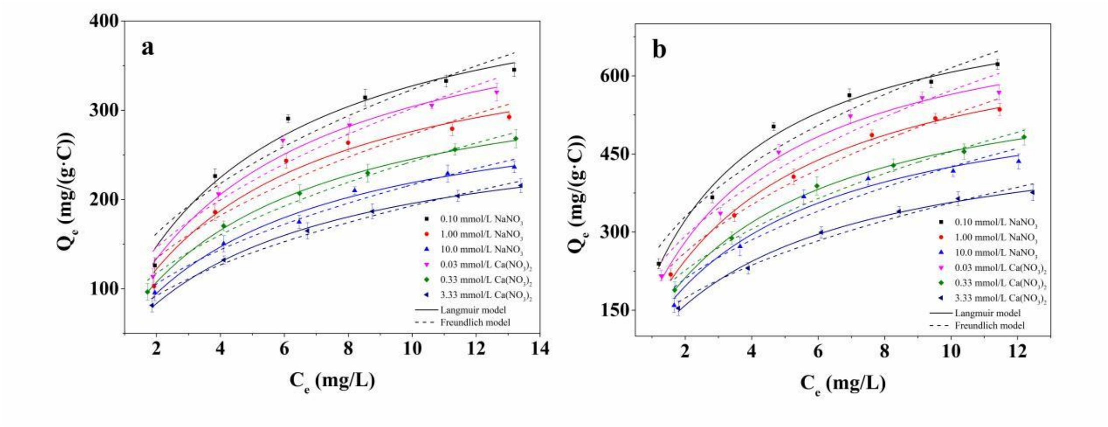

Fig. S6 Adsorption isotherm curves of Pb^{2+} on HA under different condition (a. pH 3.0, b. pH 6.0)

(mean value ± SD, n=3)

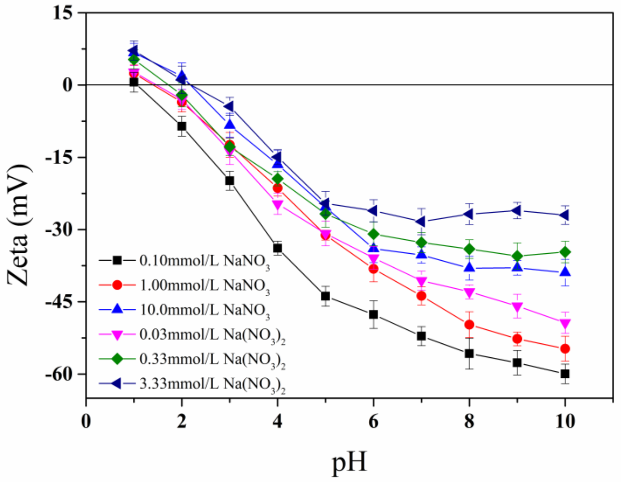

Fig. S7 Zeta potential of PSMPs under different conditions (mean value ± SD, n=3)

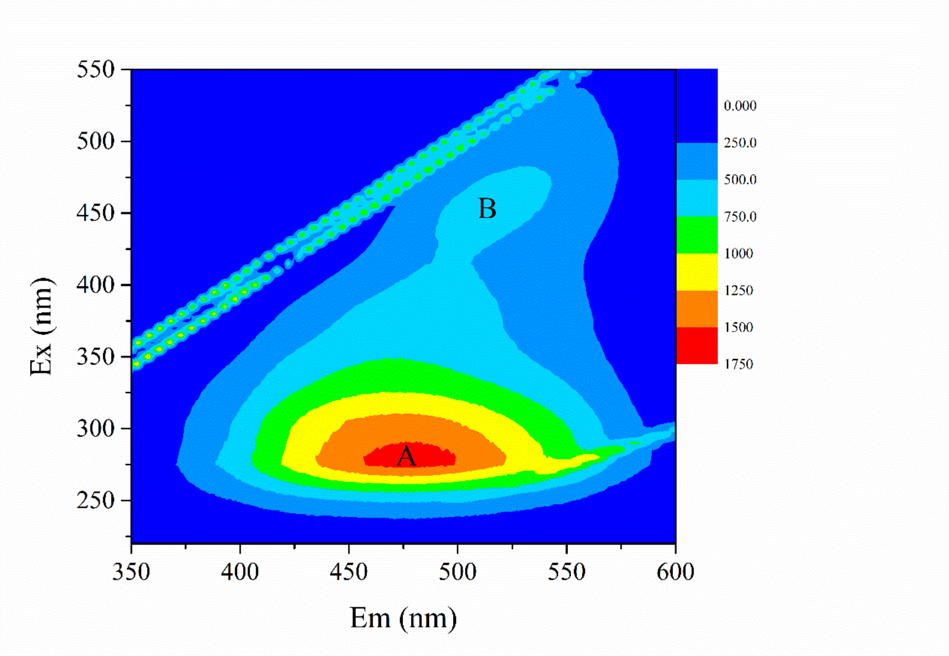

Fig.S8 The three-dimensional fluorescence excitation-emission matrix(3D-EEM) of HA

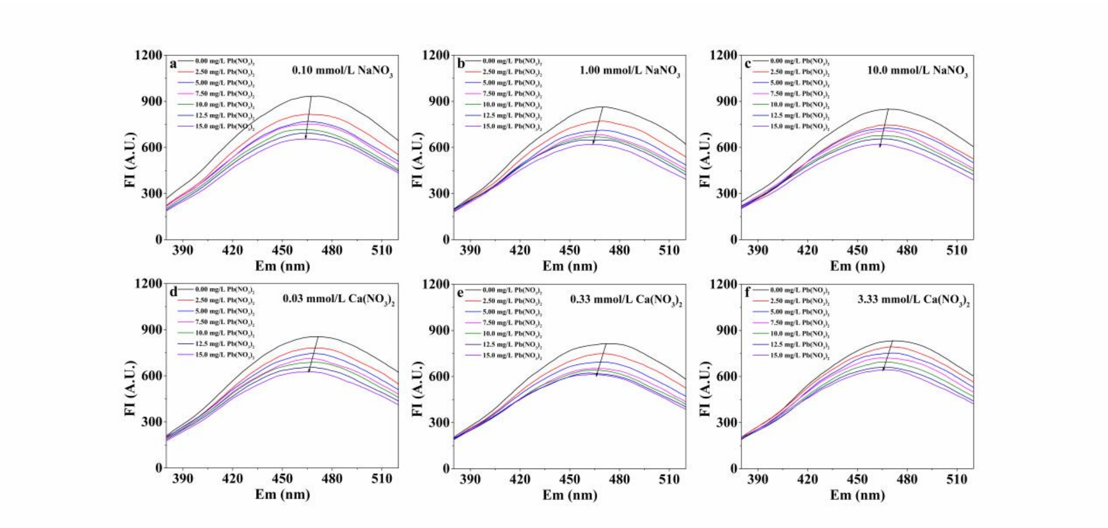

Fig. S9 Fluorescence spectra of HA with increasing Pb^{2+} concentrations in HA-Pb^{2+} system

(pH 3.0, 5.00mg·C/L HA)

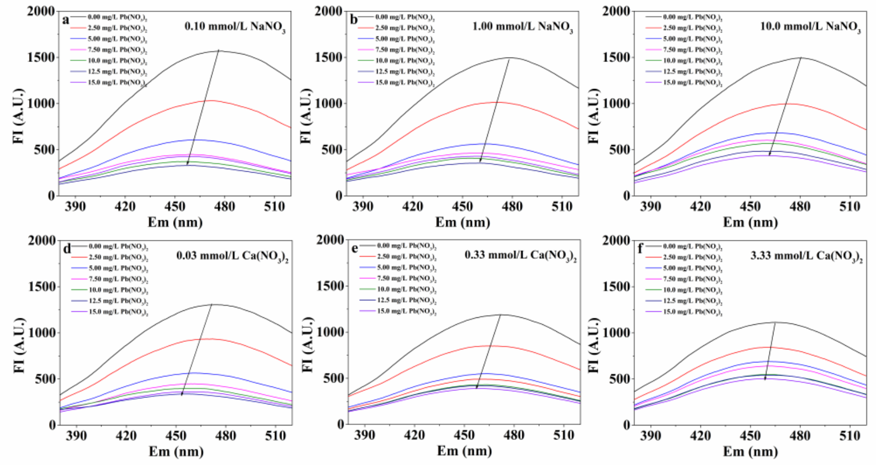

Fig. S10 Fluorescence spectra of HA with increasing Pb^{2+} concentrations in HA-Pb^{2+} system

(pH 6.0, 5.00mg·C/L HA)

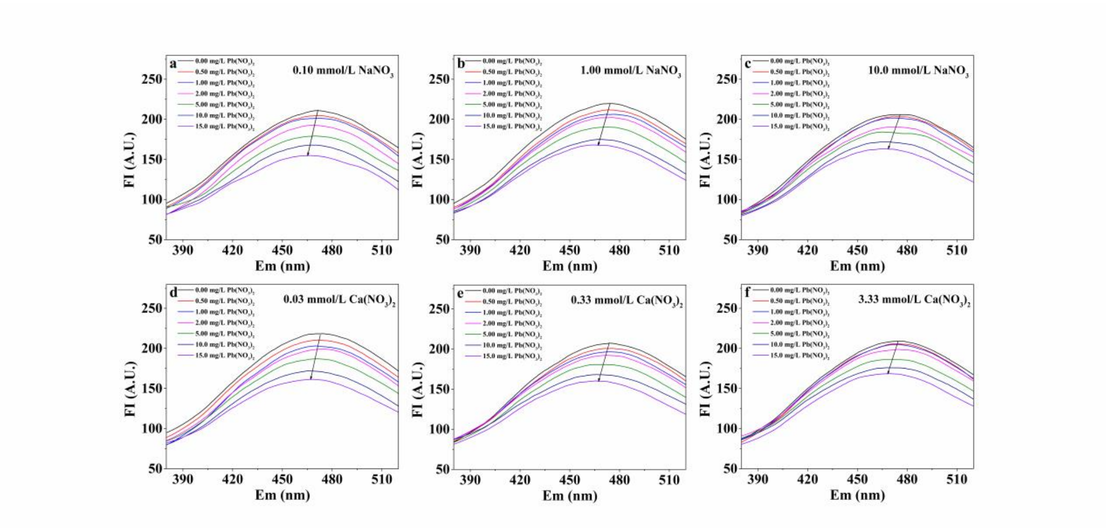

Fig. S11 Fluorescence spectra of HA with increasing Pb^{2+} concentrations in PSMPs-HA-Pb^{2+} system (pH 3.0, 1.00mg·C/L HA)

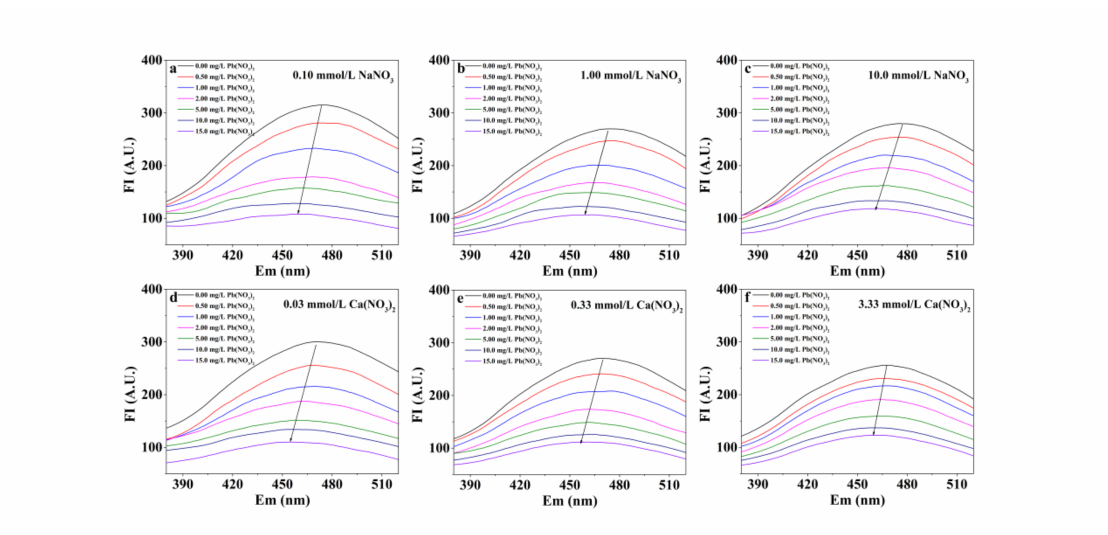

Fig. S12 Fluorescence spectra of HA with increasing Pb^{2+} concentrations in PSMPs-HA-Pb^{2+} system (pH 6.0, 1.00mg·C/L HA)

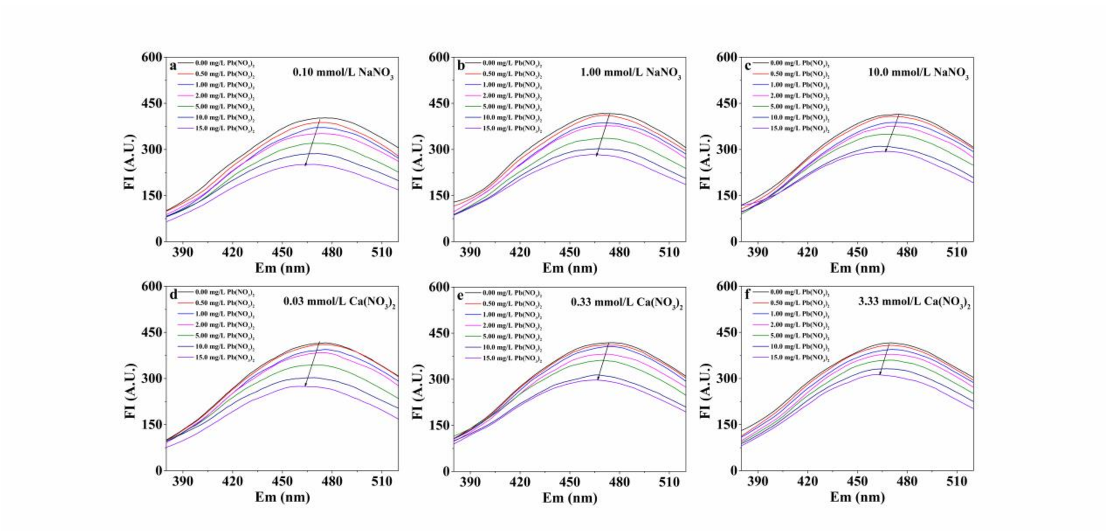

Fig. S13 Fluorescence spectra of HA with increasing Pb^{2+} concentrations in PSMPs-HA-Pb^{2+} system (pH 3.0, 2.50mg·C/L HA)

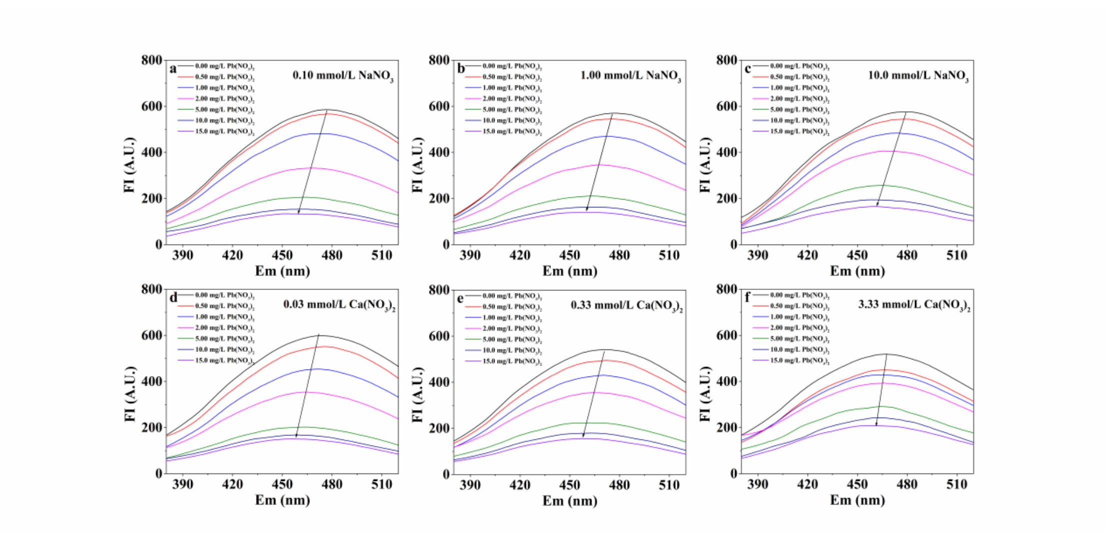

Fig. S14 Fluorescence spectra of HA with increasing Pb^{2+} concentrations in PSMPs-HA-Pb^{2+} system (pH 6.0, 2.50mg·C/L HA)

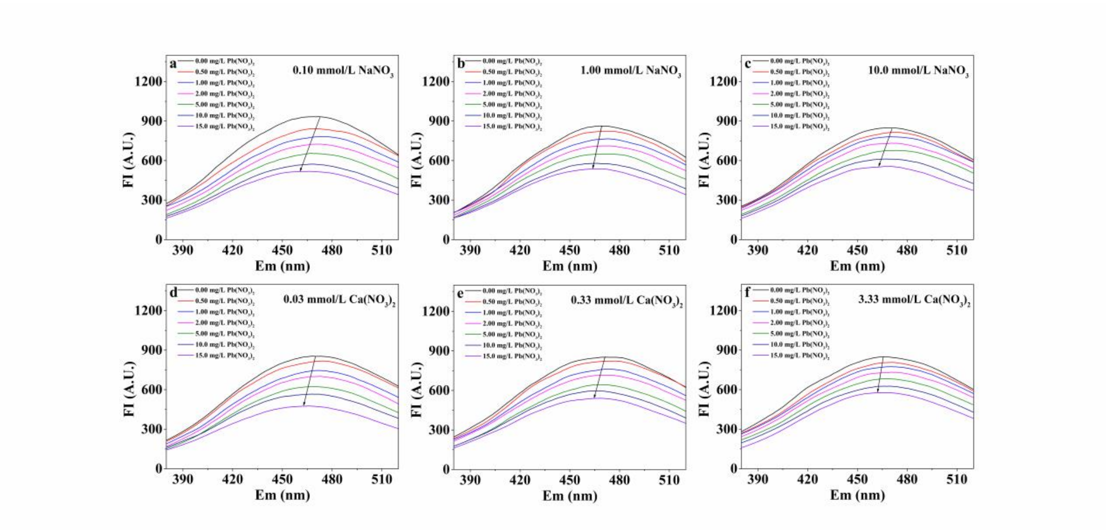

Fig. S15 Fluorescence spectra of HA with increasing Pb^{2+} concentrations in PSMPs-HA-Pb^{2+} system (pH 3.0, 5.00mg·C/L HA)

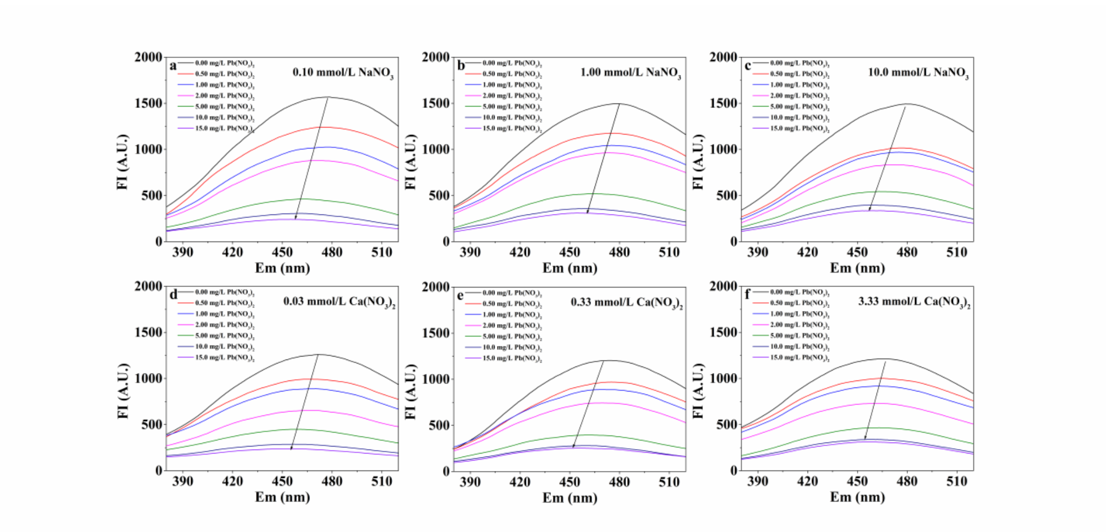

Fig. S16 Fluorescence spectra of HA with increasing Pb^{2+} concentrations in PSMPs-HA-Pb^{2+} system (pH 6.0, 5.00mg·C/L HA)

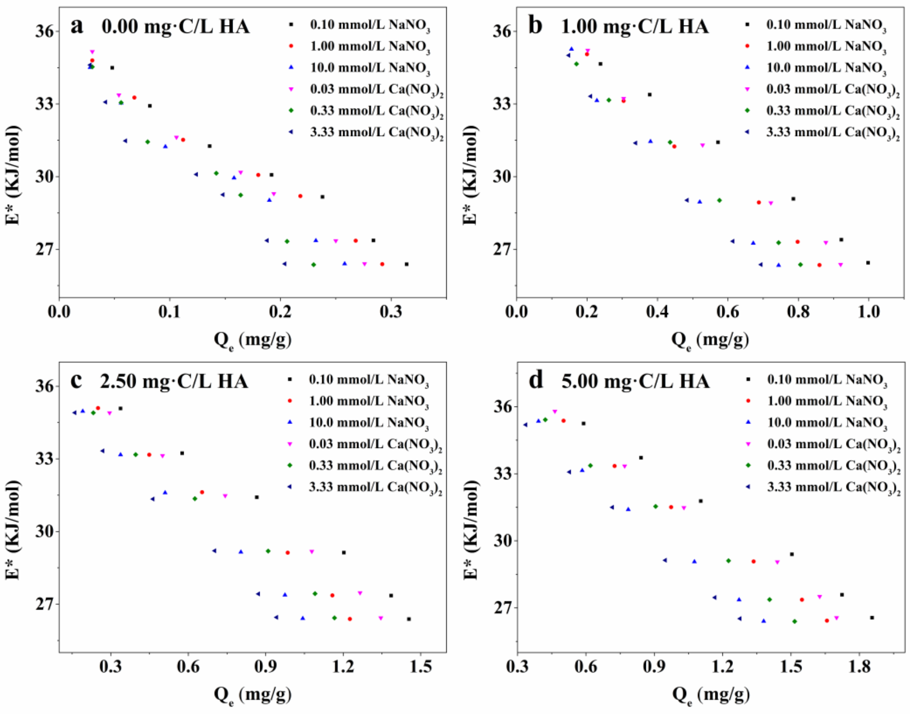

Fig. S17 Site energy (E^*) of PSMPs against Pb^{2+} adsorption capacity (Q_e) at pH 3.0

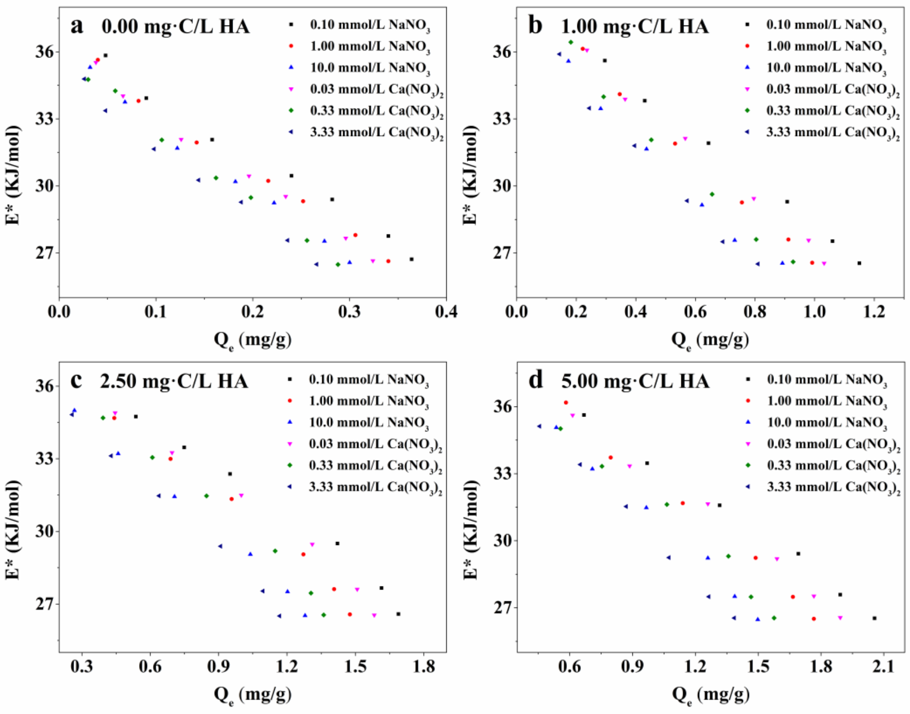

Fig. S18 Site energy (E^*) of PSMPs against Pb^{2+} adsorption capacity (Q_e) at pH 6.0
